# Rice straw for energy and value-added products in China: a review

**DOI:** 10.1007/s10311-023-01612-3

**Published:** 2023-06-15

**Authors:** Ahmed Alengebawy, Yi Ran, Nirmal Ghimire, Ahmed I. Osman, Ping Ai

**Affiliations:** 1 College of Engineering, Huazhong Agricultural University, Wuhan, 430070 China; 2 Biogas Institute of Ministry of Agriculture and Rural Affairs, Chengdu, 610041 China; 3 Department of Chemical Science and Engineering, Kathmandu University, Dhulikhel, 44600 Nepal; 4 School of Chemistry and Chemical Engineering, Queen’s University Belfast, David Keir Building, Stranmillis Road, Belfast, BT9 5AG Northern Ireland, UK; 5 Technology & Equipment Center for Carbon Neutrality, Huazhong Agricultural University, Wuhan, 430070 China

**Keywords:** Rice straw, Biorefinery, Biogas production, Biofertilizer, Life cycle assessment, Environmental impact assessment

## Abstract

The rise of global waste and the decline of fossil fuels are calling for recycling waste into energy and materials. For example, rice straw, a by-product of rice cultivation, can be converted into biogas and by-products with added value, e.g., biofertilizer, yet processing rice straw is limited by the low energy content, high ash and silica, low nitrogen, high moisture, and high-quality variability. Here, we review the recycling of rice straw with focus on the global and Chinese energy situations, conversion of rice straw into energy and gas, biogas digestate management, cogeneration, biogas upgrading, bioeconomy, and life cycle assessment. The quality of rice straw can be improved by pretreatments, such as baling, ensiling, and co-digestion of rice straw with other feedstocks. The biogas digestate can be used to fertilize soils. The average annual potential energy of collectable rice straw, with a lower heating value of 15.35 megajoule/kilogram, over the past ten years (2013–2022) could reach 2.41 × 10^9^ megajoule.

## Introduction

As in the rest of the world, the majority of China’s energy consumption comes from fossil fuels, particularly coal (Ahmed 2023). Hence, significant carbon dioxide and greenhouse gas emissions are generated from the combustion of these fossil fuels (Kang et al. 2020). As a result of China’s significance in deciding the stability of the global climate, numerous mitigation initiatives have been implemented. China included energy and carbon intensity benchmarks mandated by the five-year plans (Liu et al. 2021b). Therefore, more sustainable alternatives, such as biomass, must be utilized to overcome these concerns (Osman et al. 2021b; Dutta et al. 2023). In addition to biomass being utilized for bioenergy production, waste is collected, transported, and possessed with comparable ease to conventional fossil fuel processing (Osman et al. 2023a). Hence, the Chinese government has undertaken several significant initiatives, including the construction of clean energy systems, such as bioenergy facilities depending on agricultural and other types of biomass (Clare et al. 2016; Chen et al. 2022).

With approximately 200 billion tons per year, lignocellulosic biomass, i.e., rice straw, is one of the most pervasive bioenergy resources on the planet. This quantity represents a substantial substrate for biofuel production (Sharma et al. 2022). Furthermore, using rice straw does not affect the food chain because rice straw is an inedible component. Therefore, utilizing this waste for energy production is essential to achieving renewable energy goals (Londoño-Pulgarin et al. 2021). China, an agriculture-based country, produces over 1.04 billion tons of agricultural waste annually, which is almost one-third of the global yield (Liu et al. 2021a). Considerable amounts of this waste, which has a high energy potential, are lost through disposal or direct burning in open fields, resulting in negative environmental impacts from fine particulate matter as well as elemental and organic carbon (Su et al. 2021). Therefore, these amounts should be utilized sustainably to generate clean bioenergy.

**Fig. 1:**
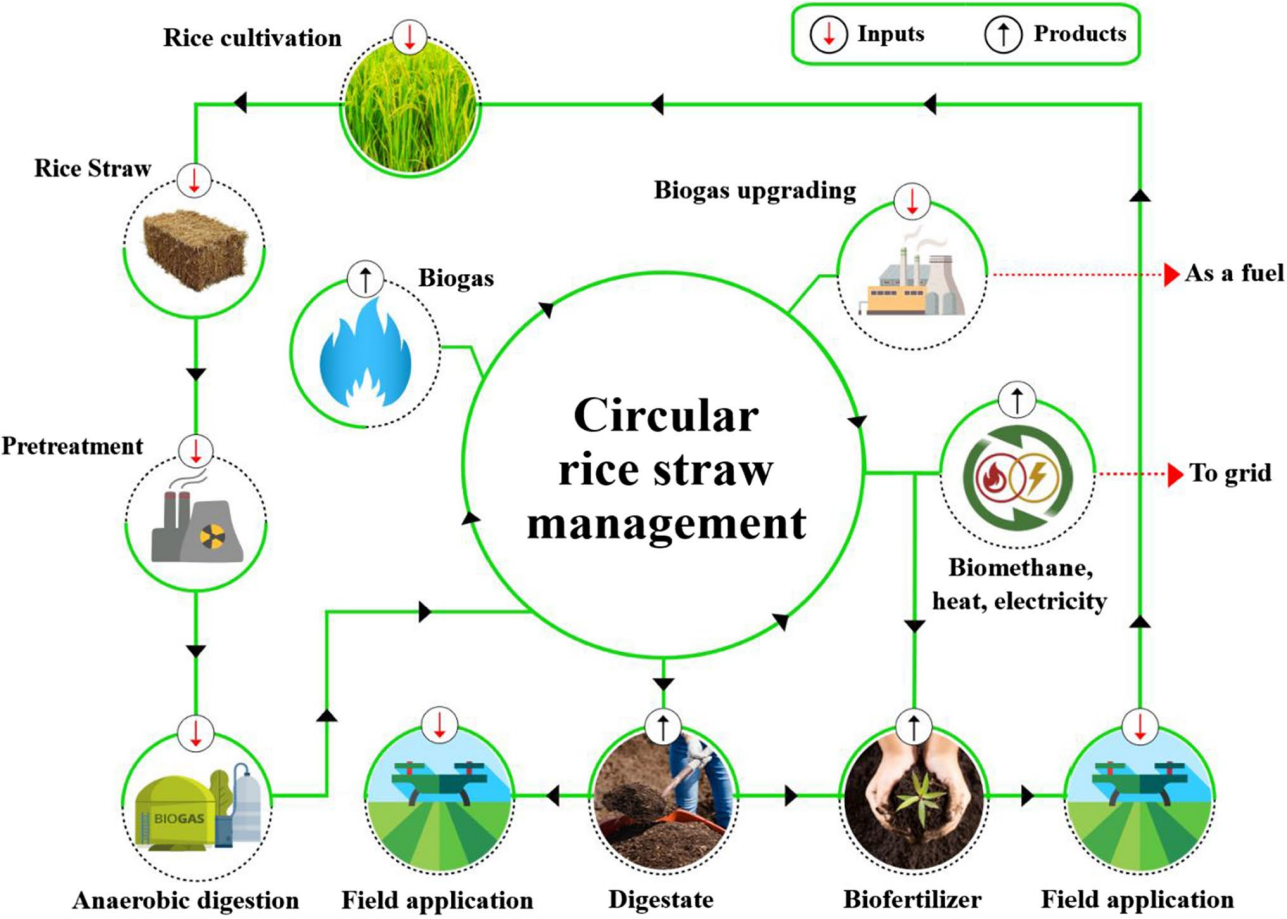
Pathways of rice straw management as a sustainable source for bioenergy and value-added by-products. Using rice straw to produce biogas is one of the most popular methods in China. Biogas can then be used to produce various forms of clean energy. In order to achieve the principles of sustainability and circular bioeconomy, the digestate resulting from the anaerobic digestion process is used to produce biofertilizers

Accordingly, rice straw was chosen as the focal point of the present study, with the primary objective of providing an overview of the appropriate use as biomass for biogas production (Alengebawy et al. 2022c). In addition to the simultaneous valorization of generated digestate as a biofertilizer and the utilization of raw biogas for electrical and thermal energy production (Farghali et al. 2022). To the best of the authors’ knowledge, the present study discusses the above-mentioned integrated approach of rice straw utilization for the first time, coupled with the environmental impact assessment, to achieve sustainability considering the circular bioeconomy principle. Figure 1 summarizes the main layout of the current review, indicating the possible integrated approaches of rice straw utilization in light of the bioenergy concept.

## Conventional energy and related emissions

### Global energy

Currently, fossil fuels constitute the predominant energy source globally, accounting for over 80% of the world’s energy supply (Hassan et al. 2021a). Extracting, transporting, and combusting fossil fuels results in the emission of substantial quantities of greenhouse gases, such as carbon dioxide, methane, and nitrous oxide (Bruhwiler et al. 2021). The emissions mentioned above are recognized to be a major contributing factor to climate change, air pollution, and various environmental and health concerns (Osman et al. 2021a; Liu et al. 2021b). Climate change and the rapid depletion of non-renewable energy sources are two of the most significant issues facing the modern economy in light of this development (Sharma et al. 2022). Increased economic activities, deforestation, and burning of fossil fuels have all been identified as major contributors to rising atmospheric pollutant gas levels (Antar et al. 2021).

Over the past few years, there has been a persistent increase in global energy consumption, primarily driven by an escalating need for energy in emerging economies (Londoño-Pulgarin et al. 2021). The International Energy Agency reports a 4.6% rise in global energy demand in 2021, with developing countries being the primary drivers of this increase (IEA 2021a). The burning of non-renewable energy sources is the primary cause of greenhouse gas emissions on a global scale, constituting roughly 73% of the overall emissions (Olivier 2020). A study by Ragazzi et al. (2017) stated that electricity and heat generation is the primary source of carbon dioxide emissions in the European Union. During the past decade, emissions from fuel energy production in the European Union reached one billion tons of carbon dioxide, accounting for 24% of carbon dioxide emissions.

Regarding the emission sources, in 2021, the international energy agency reported that non-renewable resources would emit 10.5 gigatons of carbon dioxide emissions from coal, 3.2 gigatons of carbon dioxide from natural gas, and 0.70 gigatons of carbon dioxide from oil (IEA 2021b). Moreover, the energy and transportation sectors are the two biggest carbon dioxide emissions-causing sectors, contributing significantly to 14.3 gigatons (38%) and 7.6 gigatons (20.2%) of worldwide carbon dioxide emissions, respectively (Statista 2021). Carbon dioxide emissions, a primary contributor to greenhouse gases (Osman et al. 2021a), are the dominant driver of climate change on a worldwide scale (Kang et al. 2020). Global emissions must be drastically reduced to avoid the worst effects of climate change (Osman et al. 2023b). Moreover, carbon dioxide emissions have a linear relationship with the increase in global temperature rise, accounting for the vast majority of all greenhouse gas emissions (Bacenetti et al. 2013).

A previous study expected that a mere 2 °C increase in the global average temperature would lead to the extinction of millions of species and other natural disasters (Sharma et al. 2022). In order to limit surface temperature rise to 1.5 °C above pre-industrial levels and mitigate the severe effects of climate change, renewable energy sources should be well utilized (Fawzy et al. 2022; Osman et al. 2023a). Nevertheless, these renewable energy sources still form a minor fraction of worldwide energy. Renewable energy sources constituted roughly 11% of the overall energy consumption in 2019 (Ritchie et al. 2022). Therefore, a greater proportion of renewable energy must be incorporated into the global primary energy supply along with atmospheric carbon removal using the nature-based solution as an effective tool in climate change mitigation routes (Shafie et al. 2014; Ramírez-Arpide et al. 2018).

### Chinese energy

China is considered the most significant energy consumer globally, and the energy industry continues to rely primarily on traditional sources, including coal, oil, and gas (Rao et al. 2023). The significant dependence on traditional energy sources has led to critical ecological issues, such as atmospheric contamination, water contamination, and the discharge of greenhouse gases (Qin et al. 2018). In the contemporary era, China has undertaken noteworthy measures to tackle the above-mentioned concerns (Bleischwitz et al. 2022; Ahmed 2023). However, there remains a considerable distance to traverse in order to realize a sustainable and environmentally friendly energy infrastructure. Coal remains the primary energy source in China, which accounts for approximately 57% of the country’s overall energy consumption (Alola et al. 2022). The consumption of such an amount of coal makes the carbon neutrality race challenging.

According to the Statistical Review of World Energy, the energy consumption rate experienced a 2.1% increment, which is lower than the 3.8% average over the past decade. The energy mix of China is progressively transitioning toward more environmentally sustainable sources, as evidenced by the reduction of coal’s proportion from 58% in 2019 to 57% in 2020 (BP 2021). The demand for coal in China experienced a 3.3% increase in 2018, primarily due to heightened demand from various industries such as power, steel, construction materials manufacturing, and chemicals (Rao et al. 2023). In 2021, China represented 55% of the total coal demand rise (IEA 2021a). Despite the unexpected impact of the coronavirus disease 2019 )COVID-19(, there was a notable increase of 6.9% in the consumption of natural gas, which stands in contrast to the global trend of a 2.3% reduction in gas consumption (BP 2021).

China, the world’s largest carbon dioxide emitter, has committed to halving its carbon dioxide emissions by 2030 (Sun et al. 2021). According to Our World in Data, China recorded 10.67 billion tons of carbon dioxide emissions from fossil fuel combustion in 2020, placing China among the countries with the highest carbon dioxide emissions (Ritchie and Roser 2020). China also plays a significant role in minimizing the consequences of global climate change, but further measures and pledges are necessary to achieve decarbonization. However, China still confronts the difficulties of attaining a peak in total carbon dioxide emissions by 2030 and reaching carbon neutrality by 2060. China is simultaneously implementing carbon neutrality initiatives (Hassan et al. 2021b).

As of March 2023, the Energy and Climate Intelligence Unit reported that 123 nations had pledged to become carbon neutral, 18 countries in the in-law stage, 45 countries in the in-policy stage, 13 countries in the in-declaration stage, and 47 countries in the proposal stage (Energy & Climate Intelligence Unit 2023). China is currently in the policy stage of this challenging race with greenhouse gas emissions of 12,055 million tons carbon dioxide emissions. The Chinese government has promised to reach peak emissions by 2030 and to attain a net-zero emissions target by 2060 (Ahmed 2023). According to the Union of Concerned Scientists report (UCS 2022), in 2019, China was the largest contributor (29%) to global carbon dioxide emissions from fossil fuels. Moreover, 83% of Chinese energy is derived from fossil fuels, compared to 17% from renewable sources in 2020 (Ahmed 2023). Thus, bioenergy should receive more attention as a clean, sustainable energy source than conventional energy production methods to achieve the planned goals (Alengebawy et al. 2022c).

In summary, the present state of traditional energy sources and their associated emissions is a cause for concern. Energy from fossil fuels emits greenhouse gases, contributing to climate change. Given the worldwide implications of climate change, these issues must be addressed immediately. However, the growing use of sustainable energy sources and innovative technology to reduce emissions from existing energy sources give a reason for optimism. Thus, the transition to cleaner, more sustainable energy generation must be prioritized to mitigate climate change and ensure a sustainable future.

## Bioenergy

Bioenergy or biomass energy is an excellent candidate to substitute fossil fuel energy. Biomass is comprised of a wide variety of organic feedstocks, including agricultural and forestry waste (Sharma et al. 2020), livestock manure (Ferrari et al. 2021), energy crops (Kang et al. 2020), food waste (Tian et al. 2021), industrial wastewater (Kim et al. 2020), municipal solid waste (Kang et al. 2020), and landfill waste (Ragazzi et al. 2017). Biomass can be utilized for the generation of various forms of energy via different thermochemical, e.g., combustion, pyrolysis, and gasification (Xiao et al. 2009) or biochemical, e.g., digestion, and composting (Londoño-Pulgarin et al. 2021). Biomass could also be utilized in an integrated approach of thermochemical and biochemical in one process (Osman et al. 2021b). Bioenergy production from agricultural biomass reduces elemental carbon emissions from open-air burning by approximately 40% of the total emissions (Phairuang et al. 2019). Elemental carbon contributes significantly to global climate change due to incomplete combustion (Wang et al. 2020a).

Accordingly, using biomass to generate bioenergy reduces the consumption of fossil fuels and carbon emissions that contribute to global climate change (Londoño-Pulgarin et al. 2021). In this context, Ardolino et al. (2021) reviewed the progress in biogas production from organic waste and explained the role of biogas in greenhouse gas emissions mitigation. They reported that developed techniques could reduce environmental emissions while simultaneously achieving a high biogas production rate. Agricultural biomass, which is typically a by-product, can be used as a substitute for fossil fuel-based energy sources (Meng et al. 2020). Biomass can be used to produce clean and eco-friendly energy, e.g., biofuels, while effectively managing agricultural residue. Biofuels can be divided based on the state of matter into solid, e.g., firewood, wood pellets, and charcoal (Salehi Jouzani et al. 2020), liquid, e.g., biodiesel, bio-oil, and bioethanol (Sreekumar et al. 2020), and gaseous, e.g., biogas, syngas, and biohydrogen (Antar et al. 2021).

### Bioenergy in China

China relies heavily on fossil fuels, particularly coal, as in other urbanized nations, to meet Chinese energy needs (Kang et al. 2020). To reduce fossil fuel consumption, the Chinese government aims to increase the proportion of renewable biomass energy in the total energy mix (Sun et al. 2021). With 140 million hectares of land used in China to produce crops, it is estimated that 882.14 million tons of biomass could be generated from crop residues (Antar et al. 2021). As of 2020, China annually produces 51 terawatt hours of power supply from over 29.5 gigawatts of installed biomass-based power plants, out of which over 13.3 gigawatts are powered by agricultural residues (Zhang et al. 2021b). Biogas and biodiesel are crucial to the bioenergy scenario in China.

The energy sector, especially biomass energy, has sparked renewed interest in recent years, along with the 12^th^, 13^th^, and 14^th^ five-year plans of China. Over the last decade, numerous studies examined the function of bioenergy, particularly in rural regions focusing on agricultural residues, such as crop straws. In 2012, Jiang et al. (2012) stated that China produced around 505.5 million tons of net accessible agricultural residues annually, with an annual bioenergy potential of approximately 253.7 million tons of standard coal, representing about 7.4 exajoules/year. In 2017, Zhao et al. (2017) emphasized the importance of identifying suitable locations for straw-based energy facilities in Hubei province. They devised optimization strategies to streamline the raw material supply chain and reduce transportation expenses. Experimental results revealed that approximately half of the 34.89 million tons of agricultural straw generated annually in Hubei Province could be used to generate electricity.

In 2020, Meng et al. (2020) techno-economically evaluated three major supported types of large-scale agricultural residue utilization projects, e.g., biogas, briquette fuel, and syngas, in terms of product benefit, environmental efficiency, and by-product disposal. The results indicated that the biogas project was the best option. In 2021, B. Zhang et al. (2021a, b) calculated the potential of agricultural resources for bioenergy in China, considering soil conservation, collecting costs, and future production and management changes. According to the findings, 226 million tons of crop residues might be gathered yearly, with an estimation of producing 495 million tons in 2050. They also discovered that Henan, Shandong, and Jiangsu provinces have strong supply potential and low collecting costs, making them the optimal places for developing residue-based bioenergy production.

Giwa et al. (2020) stated that although biogas production from domestic plants is likely to decrease to 10 × 10^27^ m^3^ by 2020, production from commercial plants is also increased to 3 − 5 × 10^27^ m^3^. Moreover, boosting biogas production is a key priority of China’s energy industry sector, which seeks to produce 300 × 10^27^ biogas by 2030. Similarly, biodiesel yield in China was increased to 1.455 billion liters by 2020, with 42 new operational production plants (Duarah et al. 2022). Furthermore, Fu et al. (2021) estimated the bioethanol production in China from most major 5 feedstocks, including corn, switchgrass, cassava, forest residues, crop straw, and sweet sorghum. Their results revealed that the bioethanol yield could reach 145.42 million tons by 2030. However, given food security, agricultural residues, such as rice straw, are the most promising biofuel production feedstock (Röder et al. 2020).

### Rice straw characteristics and utilization methods

Rice straw is produced in large quantities in China, with an annual output of 230 million tons (Liu et al. 2021a). Regrettably, a significant portion of this rice straw remains unused and subsequently disposed of or burned in the open fields, resulting in ecological predicaments, such as atmospheric and aquatic contamination, in addition to soil deterioration (Alengebawy et al. 2022c). The sustainable utilization of rice straw has been promoted by the Chinese government through the implementation of diverse policies aimed at addressing the issue at hand (Ren et al. 2019). Several provinces in China have offered financial incentives for establishing power plants that utilize rice straw-based biogas technology (Sun et al. 2019). According to Shafie et al. (2014), rice straw exhibits a significantly higher heating value of up to more than 16 megajoule/kilogram, which confers upon rice straw the benefit of possessing a greater capacity for energy production. The proximate and ultimate analyses of rice straw in China are listed in Table 1.

**Table 1:** Average values of approximate and ultimate analyzes of rice straw in China.

Proximate analysis (wt.%, dry basis)	Values^a^	Ultimate analysis (wt.%, dry basis)	Values^a^
Moisture	10.78	Hydrogen	6.03
Fixed carbon	13.85	Carbon	46.65
Ash	12.09	Oxygen	41.75
Lignin	18.83	Sulfur	0.23
Cellulose	38.44	Nitrogen	1.02
Hemicellulose	27.21	Lower heating value^b^	15.35

Characteristic values of rice straw vary from region to region due to climatic and soil conditions. However, the average characteristic values of rice straw in different regions of China, e.g., 10.78% moisture and 15.35 lower heating value, make rice straw a good candidate for sustainable use. These values are a good reference for analytical studies and those interested in the sustainable use of rice straw

^a^The numbers in the table are calculated as the average values in different provinces

^b^The unit of lower heating value is megajoule/kilogram

**Fig. 2:**
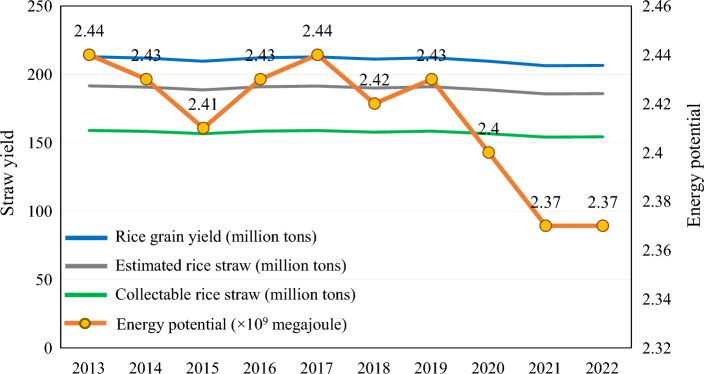
Ten-year estimates of rice straw yields and the energy potential in China. The surplus amounts of rice straw in China, in combination with other types of biomass, are sufficient to produce a substantial amount of energy and make bioenergy a strong competitor to other renewable energies. The average annual energy potential of the rice straw that may be collected is 2.41 × 10^9^ megajoule

Accordingly, the national energy yields of rice straw should be counted in order to estimate the energy potential of these yields; therefore, new sustainable approaches should be recommended to maximize the value of rice straw. The total rice straw yield was determined by applying Eq. 1 according to the national output of rice grain (NBS 2022) using the grain-to-straw ratio. The collectable straw yield was also calculated from Eq. 2 based on the collectable coefficient presented by Ai et al. (2015). Moreover, the energy potential from rice straw yield was calculated using Eq. 3 based on the average lower heating value of rice straw over China, according to the methodology presented by Jiang et al. (2012). The ten-year estimates (2013–2022) of rice straw yields and energy content are presented in Fig. 2.

1 $${Y}_{S}=\sum {Y}_{G}\times R$$

2 $${Y}_{CS}=\sum {Y}_{S}\times C$$

3 $$EP=\sum {Y}_{CS}\times LHV$$

where *Y*_*S*_ is the yield of rice straw, *Y*_*G*_ denotes the yield of rice grain, *R* is the average grain-to-straw ratio (0.9) over China, *Y*_*CS*_ refers to the yield of collectable rice straw, *C* is the collectable coefficient (0.83), *EP* refers to the energy potential, and *LHV* is the lower heating value of rice straw, expressed as 15.35 megajoule/kilogram (Table 1).

**Fig. 3:**
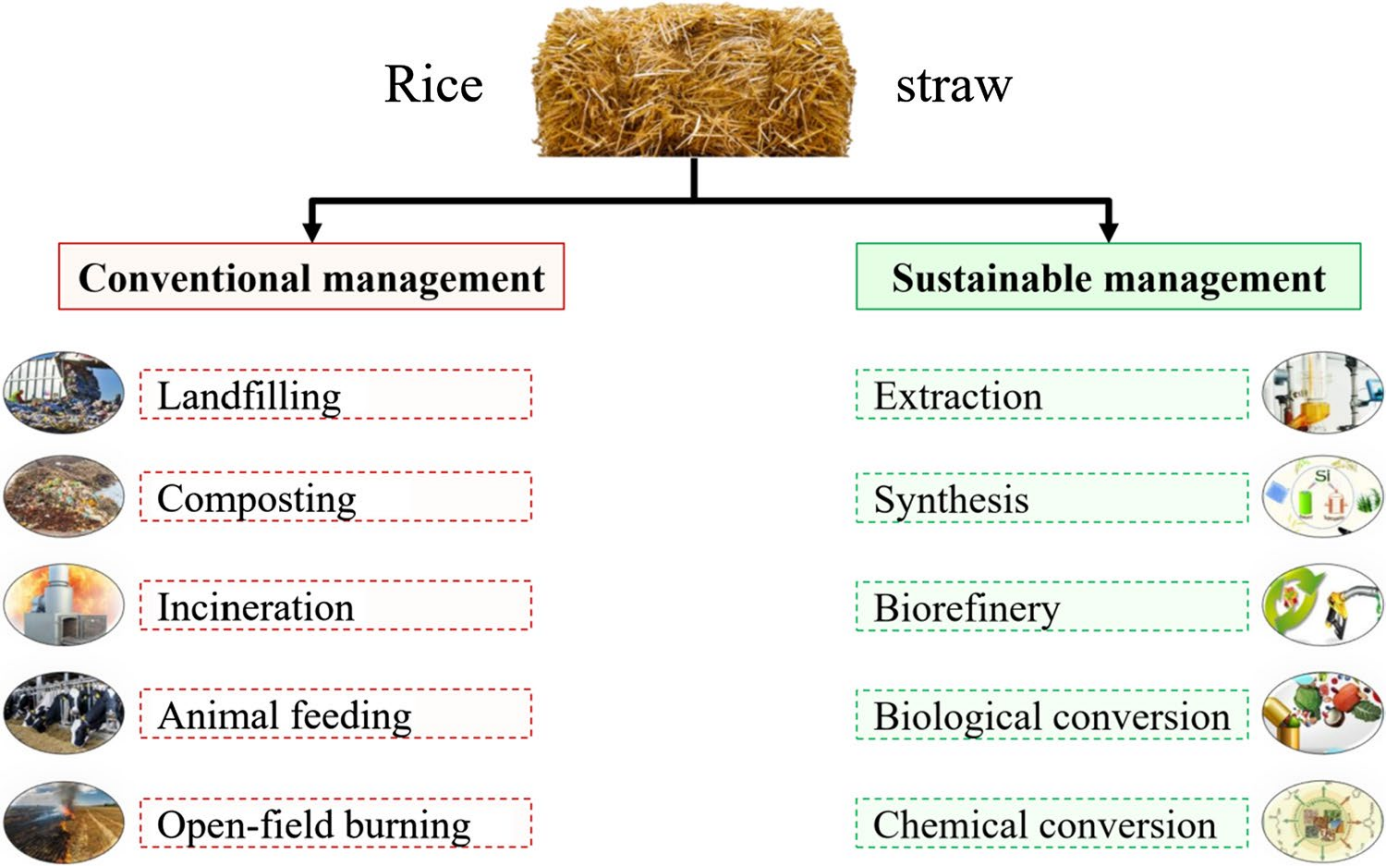
A comparison of conventional and sustainable management of rice straw for producing biofuels and value-added products. Conventional management of rice straw ends up having negative impacts on the environment and the whole earth. On the other hand, sustainable management of rice straw is an alternative approach and one of the promising methods for rescuing the planet from climatic disasters

The collectable amount of rice straw has a high average energy potential, reaching 2.41 × 10^9^ megajoule annually. Therefore, these abundant amounts should be used to produce clean energy instead of direct burning that pollutes the environment and affect the entire ecosystem (Alengebawy et al. 2022c). Furthermore, the yield of rice and rice straw, along with the energy content, decreased in 2020–2022 due to the direct and indirect implications of the coronavirus disease (COVID-19) crisis, e.g., unavailability of labor. However, agricultural production is expected to rise again, with life gradually returning to normal. In conjunction with this point, we can emphasize that the conventional methods of using rice straw should be replaced with promising sustainable approaches that could achieve a circular bioeconomy (Yang et al. 2023) since the current conventional management of rice straw had a negative impact on the climate change and global warming potential. A comparison of conventional and sustainable rice straw management methods is illustrated in Fig. 3.

**Fig. 4:**
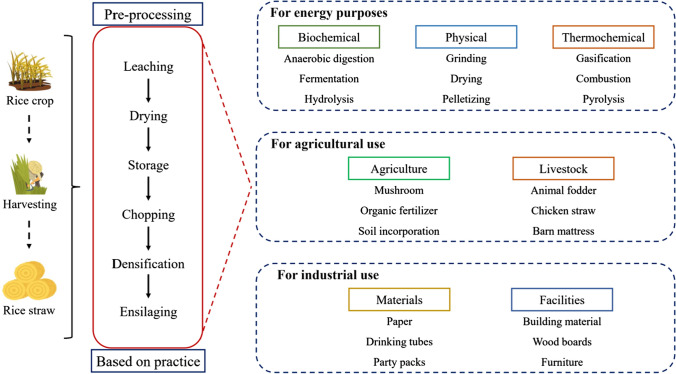
Most common rice straw utilization methods in different sectors. Rice straw can be used as feedstock in different fields, not only for biorefinery approaches but also for other uses. For example, rice straw can be used as a material for agricultural purposes, such as mushroom cultivation, soil incorporation, and animal fodder. Moreover, rice straw can be involved in the industrial sector as a building material and various paper products

Rice straw is typically utilized for (i) energy production through various conversion techniques, (ii) agricultural purposes, and (iii) industrial applications. However, the rice straw must be preprocessed or pretreated for certain applications, including drying, chopping, densifying, and others. Figure 4 provides additional examples of these practices. However, this review focuses solely on rice straw utilization for energy, as rice straw is the most prevalent and significant application.

### Rice straw utilization for bioenergy production

Rice straw has physical and chemical properties, which is a strong competitor with other biomasses for bioenergy production. Rice straw has an average calorific value of 14–15 megajoule/kilogram. Additionally, rice straw has a high volatile matter concentration (60–70%), which makes rice straw comparable to other types of biomass (Van Hung et al. 2020). China and India together account for around 53% of global rice output (Sharma et al. 2020). China produces around 270 million tons/year of rice straw, and 30% of this amount is burnt in the open fields (Xia et al. 2018). This burning releases harmful compounds, such as nitrous oxides, carbon monoxide, and volatile organic compounds (Sharma et al. 2020). High silica content makes rice straw unsuitable for cattle feed, forcing farmers to open-field burning (Van Hung et al. 2020). However, rice straw could be used to create value addition by producing different types of biofuels, such as biogas, bioethanol, briquette fuel, bio-oil, and syngas (Alengebawy et al. 2022c), in addition to value-added chemicals, such as acid–hydrolyzed furfural, furans, and furfural (Kumar et al. 2021).

**Fig. 5:**
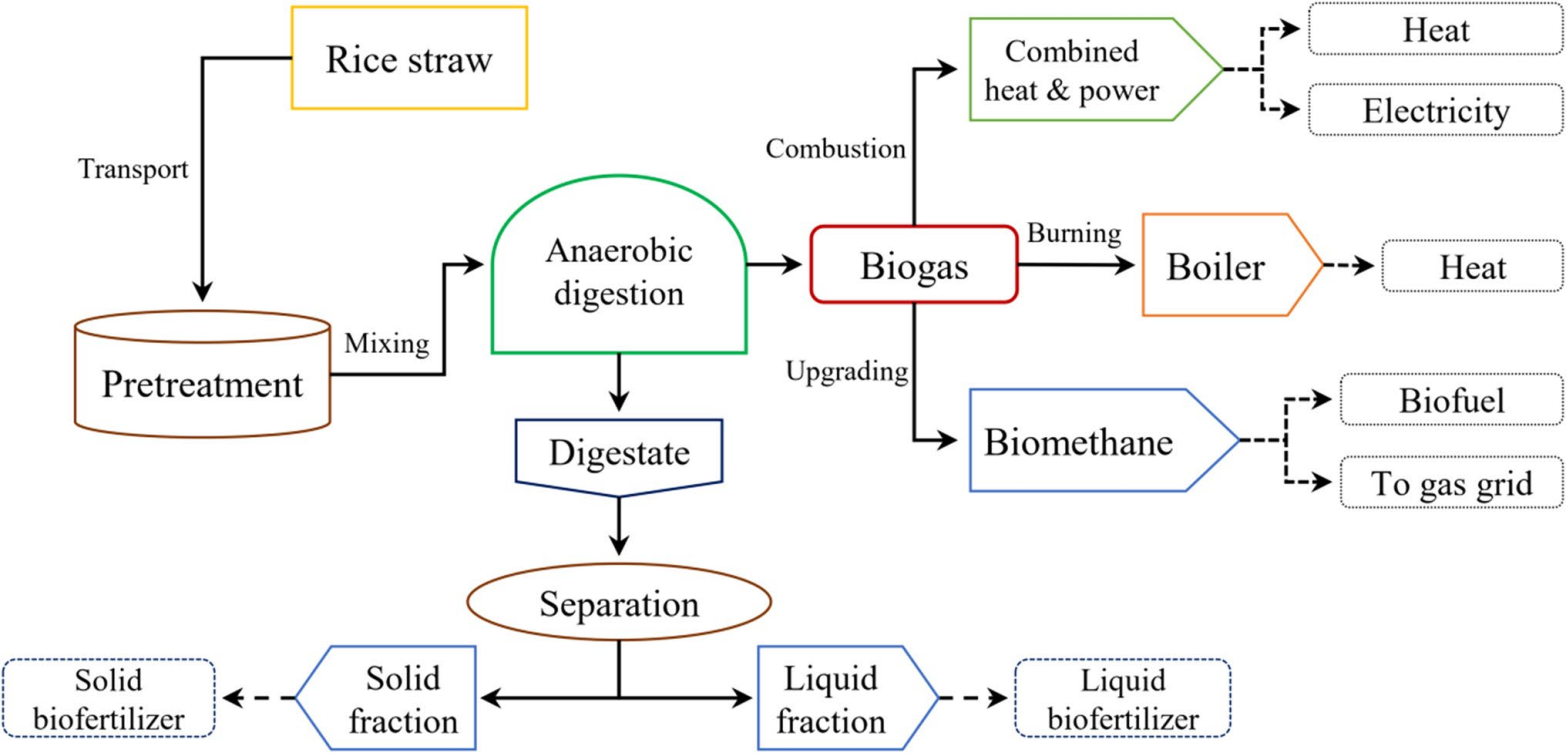
Rice straw in a biorefinery approach. One of the most common rice straw utilization schemes in China is the production of biogas by anaerobic digestion. Then, the biogas produced is used in several applications, such as combustion, upgrading, and compression. The remaining digestate is usually separated into solid and liquid fractions for further use as a biofertilizer or other applications

Biogas is a renewable gaseous biofuel replacing natural gas in various countries, including China (Sun et al. 2021). As biogas is a mature technology, Liu et al. (2016) estimated the theoretical biomass-based biomethane yield in China as 888.78 billion cubic meters, while the practical yield reached 316.30 billion cubic meters, which may subsequently be utilized to generate energy and heat. Anaerobic digestion may be utilized to convert rice straw into biomethane as clean energy. Syngas is another type of gaseous biofuel produced by gasification and can be purified and upgraded as transportation fuels, methane, dimethyl ether, methanol, and ethanol (Antar et al. 2021). In addition, bioethanol is produced as a fuel through the microbial fermentation of fermentable carbohydrates to ethanol, a process that reduces complex organic compounds to their component parts. Enzymatic hydrolysis can release mono- and fermentable disaccharide sugars, which are then converted by yeast into ethanol, carbon dioxide, and other by-products (Tse et al. 2021). With the focus on biogas, the full route of rice straw conversion into biogas and the use of biogas to produce bioenergy, in addition to biofertilizer production, is illustrated in Fig. 5.

### Rice straw-based biogas production

Efficient management of rice straws is a problem in many developing countries, such as China, India, and the Philippines, which meet their energy demand by importing fossil-based fuels (Bhattacharyya et al. 2021). Biogas from rice straw could be a suitable alternative to manage the resourceful rice straw by generating biomethane to meet local energy demands, especially in rural communities (Röder et al. 2020). Biogas is produced by the biological conversion of rice straw in an oxygen-free environment with the help of microorganisms in different steps known as the anaerobic digestion process (Atelge et al. 2020). However, some pretreatments may be required to enhance biomass digestibility and biogas yield, especially rice straw with a complex composition (Ghimire et al. 2021). Pretreatment breaks down lignin and helps enzymes easily break down high molecular weight compounds into simple sugars to enhance the anaerobic digestion process (Ferrari et al. 2021).

Several pretreatments are available for rice straw, such as physical, chemical, and biological pretreatments (Sun et al. 2021). Each pretreatment has merits and demerits and should be chosen carefully to improve methane yield and avoid downstream processing (Periyasamy et al. 2022). After the anaerobic digestion process, biogas can be combusted directly as a cooking fuel (Rajendran et al. 2013), combusted in a combined heat and power unit for heat and power generation (Yin et al. 2021), combusted in a boiler for heat generation (Kim et al. 2020), reformed for hydrogen production (Guerrero et al. 2020), or upgraded into pure biomethane (Ardolino et al. 2021). Another important process after anaerobic digestion is managing the digestate generated after biogas production (Alengebawy et al. 2022b). Anaerobic digestate can be processed via two approaches; called conventional and sustainable methods. In the following sections, we review the challenges of digestate management and the possible valorization methods. Simultaneously, the raw biogas should be valorized instead of the direct burning as a cocking fuel, which is also reviewed later after the digestate management part.

### Challenges of rice straw as a biogas feedstock

Although rice straw has many benefits over other feedstocks in producing biogas, including non-interference with food supply, low price, and relatively high biogas production, direct utilization in anaerobic digestion is limited (Dahadha et al. 2017). Recalcitrant lignocellulosic structure makes rice straw difficult to be broken down by microorganisms. This slows hydrolysis, the first rate-limiting step of the anaerobic digestion process, which subsequently leads to inefficient biogas production. Traditionally, biogas production from rice straw is based on solid-state anaerobic digestion, which is operated at a total solid content of more than 15%. Solid-state anaerobic digestion has various problems, such as inefficient biogas production, hindered mass transfer between lignocellulosic biomass and microbes, process instability, inhibition from intermediate products such as ammonia and volatile fatty acids, and problems in end-product management (Yang et al. 2015). The most common challenges of using rice straw as a biogas feedstock are summarized in Table 2.

**Table 2:** Key challenges of rice straw as feedstock for biogas production and alternative solutions

Issue	Description	Suggested solutions	References
Low energy content	In comparison with other feedstocks (e.g., energy crops), rice straw has a low energy content, which might be less effective as a biogas feedstock when used alone	Some pretreatment methods can be involved to enhance the calorific value, such as torrefaction and pelletization	Menardo et al. 2015 Bhattacharyya et al. 2021
Low nitrogen content	The low nitrogen content might inhibit the development of microorganisms during the digestion process and reduce biogas production	Mixing rice straw with another substrate that has a low carbon content is a common method to adjust the carbon/nitrogen ratio of rice straw, enhancing the biogas yield while maintaining the cost low	Atelge et al. 2020 Mothe and Polisetty 2021
High ash content	Rice straw contains a significant amount of ash, which can contribute to equipment blockage and corrosion and reduce the effectiveness of the anaerobic digestion process	Pretreatment of rice straw by washing is a promising method for removing ash; acid solution and hot water are the most commonly used methods	Chen et al. 2019
Seasonal availability	Rice straw is often only accessible during the harvesting period, which is challenging to provide a regular supply of feedstock to the biogas plant	Formation of briquettes and pellets with a suitable bulk density may be an alternative method to get rice straw in non-season times	Bhattacharyya et al. 2021 Hassan et al. 2021b
High variability in quality	The quality varies based on the type, growth environments, and storage practices of rice crops, which might impact the output energy efficacy	Recently, for example, some types of genetically modified rice have more good characteristics, which can be reflected in the properties of straw. Also, some logistical solutions for using the rice straw of each region may be a solution to reduce the conflict in the characteristics	Singh and Patel 2022
High moisture content	The high moisture content can raise drying costs and make the subsequent processes, i.e., storage, more challenging	The most common way to reduce the water content in raw rice straw is drying by sun drying or machine drying	Logeswaran et al. 2020 Shang et al. 2020

Rice straw is an abundant feedstock for biofuel production worldwide, not only in China. However, some characteristics need to be enhanced to increase the efficiency of output products while maintaining economic viability. Low energy and nitrogen contents, seasonal availability, and others obstruct valorizing rice straw efficiently. But some problems have been well addressed, and still, several developments are ongoing

### Techniques to enhance rice straw digestibility

The challenges, however, can be overcome by several methods (Mothe and Polisetty 2021). Pretreatment, such as chemical, physical, and biological, of rice straw is the most widely applied method to improve digestibility during the anaerobic digestion process. Pretreatment changes the complex structure of rice straw and overcomes recalcitrance hindering microbial and enzymatic degradation and improving hydrolysis (Hendriks and Zeeman 2009). A destructive approach such as hydrothermal pretreatment liquefies the rice straw overcoming the limitations of solid-state anaerobic digestion and enabling the change of inlet mode from solid to liquid to accommodate a wider range of efficient reactors such as high-rate sludge bed (Ghimire et al. 2021). Similarly, co-digestion with the nitrogen-rich substrate to balance the carbon/nitrogen ratio is another option to enhance the digestibility of rice straw. High carbon content and lack of macro- and micro-nutrient rice straw hinder efficient biogas production. Co-digestion supplements necessary nutrients and enhances microbial diversity, making a robust anaerobic digestion system for efficient methane production (Adarme et al. 2019).

In order to maintain the stability of biogas production from rice straw and enhance the biogas yield, rice straw must be initially pretreated through various methods, including physical, chemical, biological, or a mixture of more than one type of treatment methods (Mothe and Polisetty 2021). The physical methods include straw chopping into smaller sizes, ultrasonic, irradiation, or steam explosion treatments in order to increase digestibility by raising the surface area. However, this method requires high energy input. The chemical methods, such as acid and alkali treatments, help break down the complex compounds in straw. Nevertheless, these methods have a potential negative impact and high cost of materials. The biological methods include straw pretreatment by microorganisms, e.g., bacteria and fungi, to simplify the complex structure of biomass. Although these methods require less energy and chemical consumption, the treatment time is longer, and the microorganisms are sensitive and need ideal conditions to grow (Mothe and Polisetty 2021). Table 3 summarizes the most substantial pretreatment methods for rice straw, as well as the co-digestion with other feedstocks to improve the yield of the resulting biogas. Combined pretreatments are common use rather than a single type of pretreatment due to the higher digestibility and resulting yield of biogas.

**Table 3:** Common pretreatment methods of rice straw for biogas production in China

Province	Feedstock	Pretreatment method			Biogas yield	References
		Physical	Chemical	Biological		
Jiangsu	Rice straw	Pretreatment of rice straw with the steam explosion at a temperature range of 200 to 220 °C in 10 °C increments	X	X	The maximum rate of biogas reached 328.7 mL/g total solids (51% increase)	Zhou et al. 2016
Sichuan	Rice straw	Pretreatment of rice straw at (90, 150, 180, and 210 °C, respectively) for 15 min	X	X	The maximum yield of biogas was 306.6 mL/g total solids	Wang et al. 2018
Hunan	Rice straw	A pre-aeration treatment of rice straw for 0, 2, 4, 6, and 8 days under 25, 35, and 45 °C, respectively	X	X	The highest biomethane yield (355.3±18.7 mL/g volatile solids) was achieved at 35 °C for 2 days	Zhou et al. 2017
Jiangxi	Rice straw + pig manure	A combined pretreatment of rice straw using ultrasonic at 53 kilohertz and 350 watts for 30 min, microwave at 800 watts for 5 min, coupled with alkali treatment at 37 ℃ for 24 h	*	X	The biogas yield was increased in the case of ultrasonic and alkaline combined pretreatment by 39.41% and in the case of microwave, alkaline, and ultrasonic combined by 46.07%	Xiang et al. 2021
Nanjing	Rice straw	X	Chemical pretreatment of rice straw using calcium hydroxide, hydrogen peroxide, and ammonia–water at different times	X	Pretreatment with calcium hydroxide for 72 h had the highest biogas yield of 413.5 mL/g volatile solids, which is 45.2% higher than the control	Du et al. 2019b
Hubei	Rice straw	X	Pretreatment of sodium hydroxide solution (2%, volume/volume), with a biomass-to-solution ratio: of 10% (weight/volume) at 60 ℃ for 48 h, followed by anaerobic digestion coupled with fermentation and enzymatic hydrolysis	X	The maximum biogas yield was achieved via the anaerobic digestion of the fermentation broth resulting from rice straw fermentation without distillation (249.9 L/kg volatile solids), with a biomethane content of 79.3%	Elsayed et al. 2018
Shanghai	Rice straw + swine manure	*	Rice straw was pretreated in the following scenarios alkaline, microwave, and alkaline-microwave, then co-digested with pig manure	X	The pretreatment increased biogas yield by 25%, which could reach 355–357 L/kg total solids	Qian et al. 2019
Jiangsu	Rice straw	*	Hydrothermal and alkaline thermal pretreatments of rice straw using calcium hydroxide	X	The highest biogas reached 411.1 mL/g volatile solids, with an increase of 24.04% compared to the control	Du et al. 2019a
Henan	Rice straw	X	X	Pretreatment of rice straw using five microbial reagents, including cow manure, sheep dung, liquid consortium, straw-decomposing consortia, and biogas slurry	The highest biogas production was obtained under the sheep dung treatment (311.7 mL/g volatile solids), with an enhancement of 88.7%	Amin et al. 2021
Jiangxi	Rice straw + pig manure	X	X	Biological pretreatment of rice straw and pig manure by cellulolytic microbial consortium	The maximum methane yield could reach 0.64 L methane/day, 62.4% higher than the control	Shen et al. 2018
Tianjin	Rice straw	X	*	Combined pretreatment of biological and chemical methods using calcium oxide and liquid fraction of digestate	The combined pretreatment resulted in a methane yield of 274.65 mL/g volatile solids, indicating a 57.56% increase compared to the control	Guan et al. 2018
Zhejiang	Rice straw	*	X	Combined pretreatment of fungal (*Pleurotus ostreatus* fungus) by incubation for 10, 20, and 30 days at 28 ℃, and milling (less than 2 mm) followed by solid-state anaerobic digestion	Fungal pretreatment for 30 days, followed by milling before anaerobic digestion, resulted in 30.4% lignin removal, and the maximum methane output was 258 L/kg volatile solids	Mustafa et al. 2017

As mentioned earlier in Table 2, many of the main challenges and obstacles to using rice straw as a feedstock for biogas production are due to some characteristic issues. Therefore, rice straw can be pretreated via different methods, commonly physical, chemical, and biological methods. These treatments can enhance the straw digestibility to maintain the stability of the anaerobic digestion process while enhancing the biogas yield

^*^: indicates the method is combined with other pretreatment methods

X: indicates the pretreatment method is not used

In summary, biogas, as a foremost bioenergy source, might be used to generate heat, power, and vehicle fuel. Biogas can promote rural development, waste management, and reduced reliance on imported non-renewable energy sources, particularly when derived from non-edible biomass, such as rice straw. Biogas production must be sustainable without affecting food production or the environment. Biogas production technologies need ongoing research and development to be effective and cost-effective. Enhancing and using biogas for transportation fuel is also important.

## Biogas digestate management

The anaerobic digestion process generates a considerable quantity of digestate, which causes several environmental issues and needs adequate management to develop biogas as a sustainable energy source (Lamolinara et al. 2022). Digestate is a combination of organic matter that has been partially digested, inorganic substances, and microbial biomass. The composition and quality of the feedstock, the anaerobic technology employed, and the operating circumstances affect the digestate characteristics (Drapanauskaite et al. 2021). The management of this digestate pertains to the procedures employed in the handling and processing of the residual substances resulting from anaerobic digestion (Cathcart et al. 2021). The proficient handling and remediation of digestate hold significant importance for various reasons, such as mitigating the ecological consequences of anaerobic digestion, enhancing the standard and worth of the ultimate products, and guaranteeing adherence to regulatory standards (Logan and Visvanathan 2019).

China alone is anticipated to create 30 × 10^27^ m^3^ of biogas by 2030, creating a massive quantity of digestate and facing formidable management issues (Giwa et al. 2020). Nowadays, digestate is widely utilized in agriculture as a soil amendment or fertilizer. Odor, high humidity, volatile fatty acids, and viscosity restrict direct use in the field without treatment (Khoshnevisan et al. 2021). Also, pathogens in digestate limit the use in the agricultural field without treatment if anaerobic digestion is not under thermophilic conditions. Furthermore, Ai et al. (2020b) reported that heavy metals had been recognized as a major issue due to the accumulation in soil and plant parts, reaching humans via the food chain. Although digestate has also been used as an additive to animal feed, public acceptance and strict legislation have limited digestate application (Logan and Visvanathan 2019). Table 4 summarizes the most typical significant difficulties in traditional digestate management.

**Table 4:** Common issues of conventional digestate management

Issue	Description	Suggested solutions	References
Storage and handling	Digestate can emit an abundant amount of emissions during storage and handling; in addition, a part can be lost during the collection, loading, and transportation processes	Appropriate storage, such as using covered tanks, and proper handling of digestate are essential for preventing digestate leaching and preserving the material’s quality	Plana and Noche 2016
Nutrient imbalance	Not always an appropriate balance of nutrients is present in digestate, which might have a detrimental influence on plant development and productivity when used as a fertilizer	The nutrient imbalance issue might be solved by adjusting the carbon/nitrogen ratio of the feedstock before the anaerobic digestion, which enhances the biogas yield while maintaining the nutrient balance in the resulting digestate	Möller and Müller 2012 Xiao et al. 2022
Odor	During storage and handling, digestate can emit foul smells, which can be a nuisance to surrounding neighbors and attract pests	Covered tanks are an ideal option to limit the odor of digestate during storage and handling	Zilio et al. 2020 Jin et al. 2021
Transportation and spreading	Transporting and distributing digestate would provide logistical challenges (e.g., high liquid content), which require special equipment	Separation of digestate at the site of the biogas plant is recommended prior to the transportation by means of a screw press or decanter centrifuge to reduce the cost and other transportation issues	Drapanauskaite et al. 2021
Pathogen and weed seeds	Pathogens and weed seeds exist in digestate as a result of using antibiotics and may pollute the soil and environment when digestate is used as a fertilizer	Isolation of such pathogens, like antibiotic-resistant bacteria, is a promising method to alleviate the risk of pollution when using digestate as a fertilizer	Atelge et al. 2020
Digestate quality	Maintaining consistent digestate quality is a challenge since the qualities depend on the feedstock, digestion, and other processes	The use of high-quality substrate is an essential aspect of producing high-quality digestate. Additionally, the use of digestate quality assurance systems helps obtain high-quality digestate	Lamolinara et al. 2022
Regulations and permissions	There may be complicated rules and permission requirements for the direct application of digestate, such as the China GB Standards of Fertilizer and Commission Regulation of the European Union	It is important to abide by the regulations and permission limits set by the government by treating digestate before usage, such as heavy metals removal	Logan and Visvanathan 2019 Chang et al. 2022

Instead of landfilling and pollution issues, digestate can be used as a value-added product with some treatments. Nevertheless, digestate is outed from anaerobic digester with some unfavorable concerns, such as odor, pathogens, and storage issues. These issues limit the effectiveness of digestate processing, resulting in high costs and difficult handling

### Conventional management of digestate

Biogas digestate has been used conventionally as organic fertilizer or soil amender. The nutrient content depends on the conditions of the anaerobic digestion process and the type of feedstocks used (Möller and Müller 2012). Ammonium concentration in digestate is higher than the substrate fed as ammonium is not degraded, and organic nitrogen is converted to ammonium in the biogas digester. Thus, the ammonium applied in the field as fertilizer is converted to nitrite and then nitrate by microorganisms, which plants easily take up as a nitrogen source (Vázquez-Rowe et al. 2015; Vaneeckhaute et al. 2017). Similarly, phosphorus and potassium also remain undigested in the digester. Carbon in the digestate is mainly low in organic content and is considered an important additive in the soil and an energy source for microbes (Logan and Visvanathan 2019). After separation, the solid fraction of digestate is used to produce particleboard, bedding material for livestock, and solid biofertilizer (Lamolinara et al. 2022). The liquid fraction can also be directly used as an organic fertilizer without treatment. However, various issues, such as atmospheric pollution, ammonia and nitrous oxide emission, risk of eutrophication, and soil contamination, have raised questions regarding the direct use of digestate as fertilizer (Duan et al. 2020). Therefore, sustainable methods for digestate valorization while ensuring application safety are required.

### Sustainable methods of digestate management

The benefits of digestate as fertilizer are overshadowed by the difficulties of transportation and storage of digestate due to the high water content (Duan et al. 2020). Biofertilizer production onsite out of digestate is suitable for overcoming this problem. Struvite precipitation from digestate is an option via producing powder biofertilizer (Styles et al. 2018), but struvite has drawbacks, such as high chemical cost and demand for strict pH control (Vaneeckhaute et al. 2017). Ammonium recovery by ammonia stripping-scrubbing process utilizing the high temperature and high total ammoniacal nitrogen content in digestate is a lucrative process and produces liquid biofertilizer (Herrera et al. 2022). Ammonia gas is separated from the liquid digestate, and ammonium sulfate is formed when reacted with sulfuric acid. Alternatively, ammonium nitrate can be formed when nitric acid is used (Brienza et al. 2021).

**Fig. 6:**
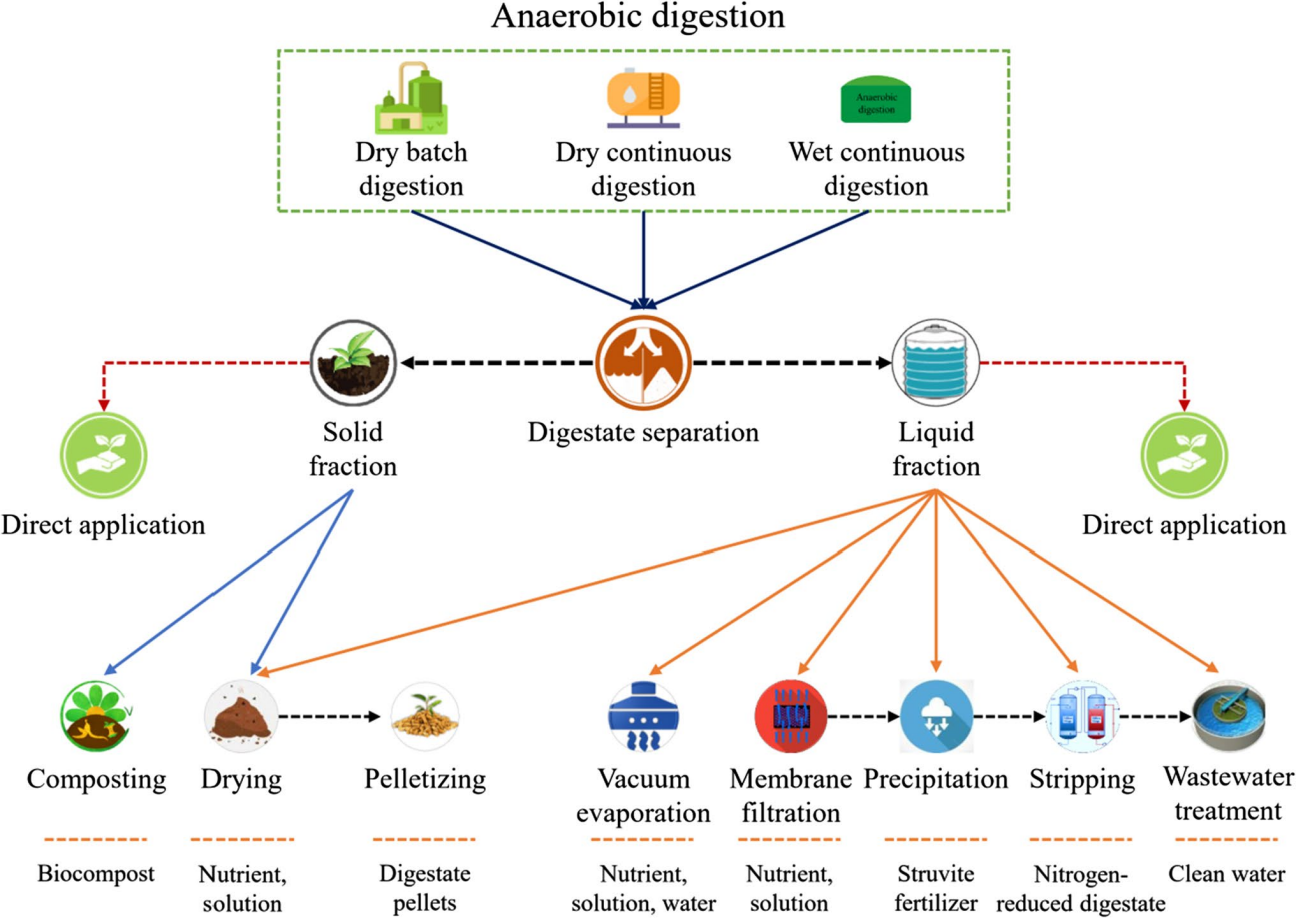
Sustainable conversion of anaerobic digestate into biofertilizer and value-added products. Digestate can first be separated into solid and liquid fractions and either applied directly or valued via different techniques. The solid fraction can be treated via composting, drying, and pelletizing. In contrast, the liquid fraction has more chances to be a feedstock for several products, such as nutrient recovery, biofertilizer production, and clean water production. This can be done via numerous techniques, i.e., evaporation, filtration, precipitation, and wastewater treatment

Moreover, the solid fraction of digestate can be further treated via drying and pelletizing (Karunanithi 2014) or biological composting (Tambone et al. 2017; Nhubu et al. 2020). Digestate drying is commercially available and a common hygienization method that produces portable and storable fertilizer (Salamat et al. 2022). Pelletizing involves compressing the raw material, reducing digestate volume while increasing the bulk density and durability of the final product (Petrova et al. 2021). Furthermore, composting solid digestate enhances digestate properties as a soil conditioner and fertilizer (Torres-Climent et al. 2015). Such marketable products can be easily transported, stored, and distributed in the field. These products help reduce mineral nitrogen and chemical fertilizers produced from non-renewable sources. Different treatment options for biogas digestate are illustrated in Fig. 6.

### Digestate solid fraction-based biofertilizer production

Digestate is separated into solid/liquid fractions, mostly by screw press separators (Cathcart et al. 2021; Brienza et al. 2021). The solid fraction contains 35% total solids, 20% total nitrogen, 30% total phosphorus, and 15% potassium (Vaneeckhaute et al. 2017). The solid fraction can be used as an organic soil amendment, but the European Union considers this fraction waste and limits the free use of digestate (Tambone et al. 2017). One of the alternatives is composting, a biological decomposition under aerobic conditions, to obtain stabilized and safe fertilizer called bio-compost (Liu et al. 2020). The imbalance between carbon and nitrogen, scarcity of carbon, and high pH limit direct composting of the solid fraction, but co-composting and additive strategies could alleviate these problems (Dsouza et al. 2021). Production of solid biofertilizer via pelletization is another alternative for producing biofertilizer pellets. Digestate is first subjected to drying to remove water to reach the desired moisture content (9–11%), then compressed in the pelletizing machine, and finally cooled the produced pellets (Cathcart et al. 2021). These pellets are then transported to the market or the field when distributed. Pellets could also be used in thermochemical processes, such as pyrolysis and gasification, to generate heat and phosphorus-rich biochar as soil amender (Antar et al. 2021).

### Digestate liquid fraction-based biofertilizer production

The liquid fraction of digestate is rich in organic matter and nutrients, such as nitrogen and phosphorus. Direct application of this fraction results in leaching these nutrients into the groundwater, causing eutrophication (Ten Hoeve et al. 2018). Biofertilizer production from this fraction is getting broad interest in capturing valuable nutrients. At the same time, as microalgae cultivation is expensive due to high nutrient costs, thus the use of the liquid fraction as a biofertilizer can reduce the cost to allow full-scale application, and the liquid fraction can also be used as media for microalgae cultivation (Duan et al. 2020). Similarly, vacuum evaporation on liquid fraction easily transported concentrated nitrogen and phosphorus content, while ammonia-stripped liquid digestate can be used as irrigation water to improve soil conditions with low nitrogen and phosphorus content (Guilayn et al. 2020). Ammonia is also stripped from the liquid fraction of digestate to produce ammonium sulfate crystals by scrubbing in sulfuric acid, which can be sold as a biofertilizer. Recently, struvite production has been considered for ease of application, transportation and storage, and no odor and pathogen contamination (Vaneeckhaute et al. 2017; Lin et al. 2018). Struvite recovered from digestate can be applied directly on the land and has a lower concentration of radioactive and heavy metals than ore-based fertilizer (Guilayn et al. 2020).

In summary, biogas digestate management is crucial to biogas production’s sustainability and environmental benefits. Biogas digestate is a nutrient-rich resource for crops and soil, but improper management may harm the environment and human health. Biogas digestate can be divided into liquid and solid parts, chemically and biologically cleaned, and used to make better biofertilizers or soil changes.

## Biogas for energy production

**Fig. 7:**
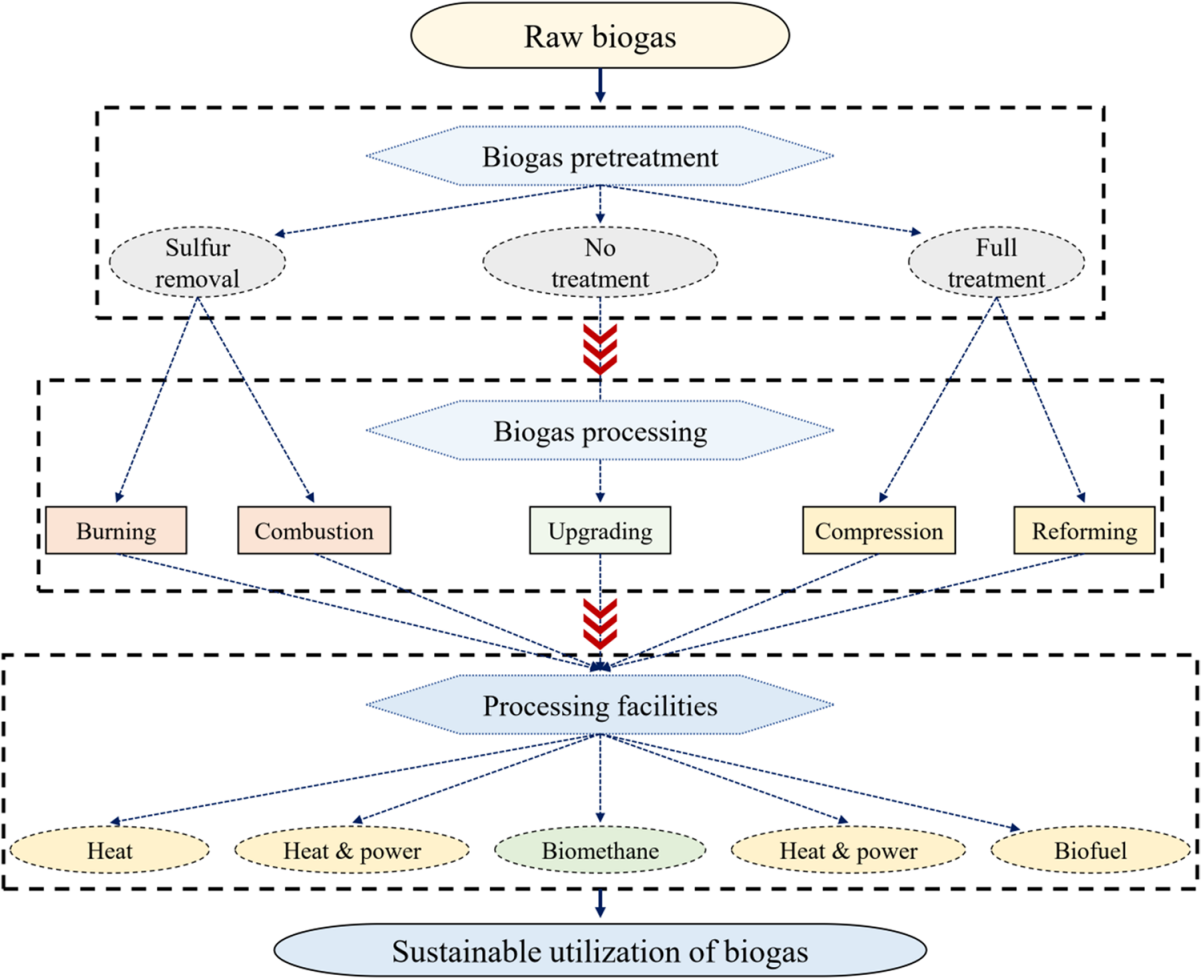
Sustainable biogas utilization pathways for bioenergy production. Here is a schematic diagram of biogas utilization methods to produce various forms of clean energy, including heat, electricity, and pure fuels. Biogas can be treated in different methods using in order to obtain a higher degree of purification according to the subsequent process, such as hydrogen sulfide removal and carbon dioxide separation. Then, biogas can be subjected to burning, combustion, or upgrading through various facilities to obtain the final product

Just as it is required to value digestate and produce value-added products, the use of raw biogas is also necessary. The raw biogas generated by the anaerobic digestion process has the potential to be converted into several forms of bioenergy. Moreover, biogas can be an attractive choice and has significantly higher energy efficiency than fossil-based fuels (i.e., coal) (Londoño-Pulgarin et al. 2021). Biomass, such as agricultural straws, can be used to generate biogas, which can be combusted to generate heat and electricity and even be used as gasoline when upgraded (Lu and Gao 2021). Biogas is converted to heat and power in internal combustion engines and power turbines on different scales. Biogas can also be combusted in boilers to generate heat (steam or hot water) and use this heat in different applications (i.e., district heating, tank heating, and steam turbines) (Parajuli et al. 2014; Kim et al. 2020). Biogas upgrading is also a technique to increase methane purity to be used as vehicle fuel or injected directly into the natural gas grid. Fuel cells are also recently emerging as an alternative due to their high efficiency, low pollutant emissions, and low noise (Wasajja et al. 2020). Therefore, dependence on fossil fuels is largely reduced when biogas is produced efficiently (LePoire and Chandrankunnel 2020). The most common biogas utilization methods are presented in Fig. 7.

### Biogas to energy by cogeneration

Biogas can be used to produce combined heat and electricity in the combined heat and power unit. The combined heat and power simultaneously produce thermal and electrical energy efficiently, reliably, and cleaner. The efficiency of combined heat and power units reaches up to 90% compared to stand-alone efficiency of 20–45% and 60% of electricity and heat generation (Abanades et al. 2021). However, water vapor and hydrogen sulfide of biogas must be removed before using biogas in engines to avoid condensation and corrosion (Cao et al. 2021). Various combined heat and power technologies, such as fuel cells, internal combustion engines, micro-turbines, and Stirling engines, use biogas for heat and electricity production. A typical combined heat and power unit consists of a reciprocating engine or combustion turbine to burn clean biogas with a heat recovery unit to convert excess heat into thermal energy (Bartocci et al. 2018).

Three combined heat and power systems were evaluated and compared by Yin et al. (2021), including reciprocating internal combustion engines, boiler/steam turbines, and micro-turbines. Their results stated that reciprocating internal combustion engines and micro-turbine systems produced lower emissions than boiler/steam turbines. Recently, fuel cells have been gaining interest in combined heat and power due to their high efficiency and less pollution. Solid oxide fuel cell is the most attractive fuel cell operating at a higher temperature of 700–1000 °C for the combined heat and power-based application on biogas (Wasajja et al. 2020). The combined heat and power unit can be used in various applications, such as institutions, residential and commercial buildings, municipality facilities, and industries.

### Biogas to heat via biogas boiler

Biogas, without upgrading, can be combusted directly in a boiler to produce heat with an efficiency of around 75–85% or higher (Ullah Khan et al. 2017). Combustion is one of the most common methods of biogas utilization for heat production. A biogas boiler can be adjusted from the conventional natural gas boiler by adjusting the air-to-gas ratio and enlarging the burner jets or fuel orifice. As biogas has a lower calorific value than natural gas, the flow rate of biogas should be increased in the adjusted combustor (Kaparaju and Rintala 2013). Although boilers can work when the biogas content of hydrogen sulfide is up to 1000 ppm, the coating is essential to avoid corrosion from higher hydrogen sulfide concentrations, and the working temperature should be above the dew point to avoid condensation (Cao et al. 2021). Besides being used for heat generation, the boiler can be used with other facilities, such as steam turbines, to generate power. In this context, Yin et al. (2021) studied three integrated systems, including a biogas boiler (500 kilowatts) with a back-pressure turbine. First, biogas is burned to produce steam, which is then pumped into a steam turbine to generate electricity. They also reported that all four systems were economically viable.

### Biogas to biomethane via biogas upgrading

Biogas contains carbon dioxide along with methane, which lowers the calorific value (22 megajoule/cubic meter); therefore, biogas needs to be cleaned and purified into pure biomethane (36 megajoule/cubic meter), which can be used as a vehicle fuel or injected into the gas grid (Akbulut et al. 2021). Upgraded biomethane is more economical in transportation and compression and avoids the negative effects of impurities, such as hydrogen sulfide and water vapor, on downstream equipment compared to raw biogas and has broader uses (Sahota et al. 2018). The most common available biogas upgrading technologies were reported by Sun et al. (2015), including pressure swing adsorption, membrane separation, chemical absorption, and water scrubbing. Moreover, cryogenic technology, compressing, and distillation to give more than 99% pure biomethane are emerging technologies (Florio et al. 2019). Similarly, the in situ biomethane enrichment is still under the nascent stage in which liquid sludge is recirculated from the digester to the desorption column, where the dissolved carbon dioxide is absorbed into the sludge. This technology is limited to small-scale plants where biomethane of more than 95% is not required (Sahota et al. 2018). Hybrid technologies are also under development to overcome the cons of different technologies by integrating them. Membrane gas permeation combined with pressurized waste scrubbing, cryogenic separation, and amine absorption is an example of a hybrid process (Ardolino et al. 2021).

### Challenges of biogas utilization for energy production

Biogas impurities need to be managed before considering energy production. Although nitrogen and carbon dioxide do not damage the components of the combined heat and power unit, they can dilute the fuel and hamper the performance of the engines. Removing oxygen can reduce undesirable fuel oxidation, while removing nitrogen improves the heating value. Adsorption can remove oxygen and nitrogen, but the process is difficult and requires high costs. Hydrogen sulfide, carbon monoxide, ammonia, and siloxanes have detrimental effects on engine components (Riley et al. 2020). Hydrogen sulfide, which causes corrosion, can be removed by adsorption and bacterial oxidation. Although carbon dioxide removal is not required for the combined heat and power process (but could be removed to improve the heating value of the biogas), carbon dioxide must be removed to upgrade biogas to reach the natural gas quality (Riley et al. 2020).

Ammonia removal is necessary to avoid the formation of nitrogen oxide emissions, which are strictly monitored to reduce their emission into the atmosphere. Failing to remove these impurities leads to corrosion, damage to the turbine and piston of engines, and solid depositions of silicon, oxygen, and calcium, thereby reducing the lifetime of engines, boilers, and end-use appliances (Kaparaju and Rintala 2013). Moreover, with the strict control of pollutant emissions of the engines, the emission requirements must be achieved. Hence, upgrading biogas using different methods is imperative, increasing costs. Similarly, sufficient biogas flow has to be guaranteed for the smooth functioning of the systems used in energy production.

In addition, the usage and distribution of heat need to be considered to ensure sustainable energy production from biogas. Electricity-alone installations should be preferred over combined heat and power to obtain higher economic and environmental value if the valorization of a high share of generated heat is impossible (Kusch 2015). Technological challenges, such as the compatibility of combined heat and power with existing facilities, also play a role in the system’s sustainability.

In summary, the utilization of biogas for energy production has significant environmental, social, and economic benefits. Biogas from biological waste can reduce greenhouse gas emissions and provide sustainable energy for power, heating, and transportation. Biogas may also improve waste management and rural development. Biogas production must be sustainable without affecting land usage or food production.

## Techno-economic assessment

### General description

Prior to investing in bioenergy projects, doing a techno-economic analysis is essential to establish the viability and possible returns. Techno-economic assessment is a technique used to examine the economic feasibility of a technology or process (Logeswaran et al. 2020). Techno-economic assessment entails thoroughly examining the technical and economic issues that affect the profitability and viability of a project (Meng et al. 2020). Usually, the techno-economic assessment method includes some general steps, such as process design, process modeling, equipment sizing, capital and operation cost estimation, and cash flow analysis (Elgarahy et al. 2023). In the case of the rice straw-based bioenergy project, the techno-economic assessment would analyze the project’s capacity to generate biogas, estimate the cost of constructing and maintaining the biogas plant, and calculate the project’s economic benefits based on the market value of the possible products, such as biogas, heat, electricity, and biomethane, in addition to biofertilizers.

The first stage in conducting a techno-economic analysis for rice straw biogas generation is determining the availability and quality of the rice straw feedstock, in addition to the energy potential (Alengebawy et al. 2022c), as presented in Sect. "Biogas digestate management". The quantity and quality of the feedstock impact the biogas output and profitability of the project as a whole. Thus, consideration must be given to the rice straw’s moisture content, lignin content, nutritional content, and other challenging issues. Pretreatment processes, such as chopping, crushing, and drying, can increase the quality of the feedstock but also affect the total cost (Song et al. 2016). Moreover, the biogas potential must be assessed, and this requires laboratory testing to establish the rice straw’s methane output and biodegradability. This data together may be used to evaluate the potential biogas output and the size of the biogas plant required to process a certain amount of rice straw.

The next stage is determining the biogas plant’s capital and operational expenses. This step comprises the cost of plant construction, equipment acquisition, operation, and maintenance (Meng et al. 2020). In addition, the expense of the feedstock and the transportation to the biogas plant must be addressed. The cost of energy and heat production, the cost of biogas purification, and the cost of waste disposal are other issues to consider. The project’s economic advantages must also be examined via the cash gained from the sale of biogas, the value of fertilizers and other by-products produced by the biogas plant, and any possible public subsidies or credits (Sganzerla et al. 2023). The potential hazards and unpredictability of the project, such as adjustments in substrate supply and energy price variations, must also be evaluated. Ultimately, the project’s environmental implications must be assessed, as presented by Alengebawy et al. (2022c, b, a). This environmental evaluation is done by the life cycle assessment tool, as described in the following section. This evaluation includes the reduction of greenhouse gas emissions and other pollutants caused by the production processes.

### Implementation of techno-economic assessment

Recently, numerous studies have been conducted on different bioenergy practices based on biomass conversion into biofuels and value-added products via various methods. These conversion methods evaluated techno-economically, such as anaerobic digestion (Doddapaneni et al. 2018), pyrolysis (Trippe et al. 2011), gasification (Bressanin et al. 2020), fermentation (Devi et al. 2021), and combustion (Morató et al. 2020). However, here we focus on evaluating rice straw as a feedstock for biogas production and the subsequent utilization of biogas and digestate. The following subsections present the implementation steps of techno-economic assessment in accordance with the study objectives, including biogas production, energy production, e.g., heat, electricity, and biomethane, and digestate valorization into biofertilizers.

#### Project scope definition

The first stage of conducting a techno-economic assessment of a bioenergy system is determining the project’s scope (Murthy 2021). The following crucial points are included in this step: (i) Identifying the project’s primary goal, such as energy generation in combination with waste management. (ii) Raw material/feedstock sourcing, in this instance, rice straw (Ezz et al. 2021). (iii) Identification of energy markets and by-products, as indicated by biogas production, followed by biogas utilization to generate heat, electricity, and biomethane, as well as the formation of biofertilizers from digestate (Diehlmann et al. 2019). The project scope should also account for legal requirements and environmental restrictions that may affect project implementation, such as the Chinese government’s current energy policy and five-year plans (Bleischwitz et al. 2022).

#### Feedstock analysis

Feedstock analysis is an essential stage in conducting a techno-economic evaluation. This stage entails examining the feedstock amount and quality based on the analytical investigations (Liu et al. 2023), as well as the transportation and storage needs, which affect the total cost. Nutritional content, moisture content, and the presence of pollutants are all factors to consider (Song et al. 2016). In this context, Sun et al. (2017) carried out a techno-economic study to evaluate the effect of straw collection, storage, and transportation in Henan province, China, on the total cost of power generation. They reported that the cost of 1-ton straw recorded about 265 Chinese Yuan. They also estimated that about 2.42 × 10^7^ Chinese Yuan could be saved when assuming 2 × 10^5^ tons of straw are purchased annually. The feedstock study aids in determining the possible energy production as well as the size of the plants required to process the feedstock.

#### Technical feasibility assessment

The current stage is usually involved in assessing the project’s technical viability. Analyzing the different production processes is also required, including selecting appropriate technology, i.e., integrating anaerobic digestion with combined heat and power system and digestate treatment unit, plant size and waste amount, and operating conditions, such as factors affecting production processes like temperature and pH. An analytical study conducted by Meng et al. (2020) evaluated different crop straw-based bioenergy projects on a large scale in Hubei province, China. They compared crop residue-based biogas, gasification, and briquette fuel projects in terms of technical and economic feasibility. Their results reflected that the biogas project has the highest overall profitability, especially in soil improvement, production cost, and expenditure saving, followed by briquette fuel and gasification projects. The technical feasibility analysis helps determine substrate suitability, maximum energy output, plant efficiency, and capital and operational expenses (Guares et al. 2021).

#### Capital and operational costs estimation

The construction and operational expenses of a key power plant are crucial in establishing a project’s economic viability (Murthy 2021). Capital expenditures include equipment for biogas production, utilization, digestate treatment, construction of different facilities, and equipment installation, whereas operational costs include raw materials processing and transportation, labor wages, and maintenance services. The cost of a project might vary based on the technology utilized, the size of the facility, and the location. An analytical study conducted by Wang et al. (2022) investigated a reasonable model of a straw supply chain for bioenergy generation in Northeast China. They explored the cost of different processes related to straw preparation, such as collection, raking, baling, and transportation. The results of the study revealed that the cost per ton of straw might be 172 Chinese Yuan. Also, the straw should be transported over a greater distance, resulting in a 53% rise in cost. However, the cost might be reduced through cross-regional use of machine purchase subsidies and agricultural machinery by 5 and 18%, respectively.

#### Revenue streams calculation

Several income streams may be obtained from a bioenergy plant, including the sale of biogas as a raw fuel, electricity produced from biogas combustion, heat generated from biogas burning, and by-products, such as biofertilizers produced from digestate valorization. The project’s income streams are contingent on the market demand for these items and the availability of transport and distribution infrastructure. Awasthi et al. (2020) stated that integrated bioenergy systems offer a number of benefits, including the entire use of feedstock, which reduces waste formation, and numerous income streams, which improve the economics of the process as a whole. Moreover, Xu et al. (2015) developed a regionalized net present value model based on a modeling of an actual production procedure of agricultural biomass-based molded products in order to evaluate the effects of different policy factors. They determined the net present value based on the product cost and cash flow. Among the products, agricultural briquette fuel had a starting price of 86 US dollars per metric ton. They also stated that China’s optimal product subsidy for agricultural briquette fuel ranges from 26 to 57 US dollars per metric ton, depending on the region.

#### Economic feasibility assessment

The economic feasibility analysis compares expected revenue streams to total capital and operational expenses (Murthy 2021). In other words, the economic feasibility assessment compares the obtained returns with the investment necessary to determine the bioenergy project’s viability. As such, it is a crucial procedure that provides business data, prevents losses, and contributes to more responsible choices (Velásquez Piñas et al. 2019). Several factors, including the supplies and services, the business’s market, investment projections, benefits, competition, market conditions, revenues, financial resources, and labor, must be evaluated. This process helps establish the financial viability and return on investments of the project (Sawale et al. 2020). There are also the payback duration, the internal rate of return, and the sensitivity analysis to consider.

### Recent techno-economic assessment research in China

Commercial and marketing skills have recently been needed to use straw as a material and energy source. In 2015, Anhui Province had about 2,000 straw market middlemen, and more people could enter the sector. Anhui mediators’ straw is used by power plants, Shandong paper mills, and a Jiangsu firm that makes edible fungus from rice straw (SPCEA 2015). Moreover, the long-term sustainability of straw use can be further ensured through the provision of financial support. The Administrative Committee of Xingan League in Inner Mongolia earmarked a budget of 50 million Chinese Yuan in 2015 to provide support for straw conversion into energy. The establishment of 200 straw fuel plants that are granular in nature has been finalized, with a processing capability of 5 × 10^5^ tons of straw annually. By the year 2020, the objective was to achieve a comprehensive utilization rate of 90% (IMNN 2017).

Clare et al. (2016) reported that feed-in tariff for bioenergy generated by facilities that use at least 80% biomass as feedstock is the main economic incentive for bioenergy generation from crop straws. Despite the policy’s effectiveness in stimulating power facility construction, some projects have faced major financial and technical obstacles, resulting in unsatisfactory operating performance and even discontinuation. They suggested that coal sharing in pre-existing power plants is an alternate approach for managing agricultural waste in China. Power plant operators do not benefit from co-firing since the co-firing does not qualify for Critical Power Feed Tariff help. The results also revealed that the co-firing of agricultural residues within 50 km of power plants might generate 89–117 terawatt hours of energy annually, assuming 10% coal power substitution co-firing ratios.

Furthermore, Chen et al. (2020) reviewed different bioenergy projects in terms of economic feasibility, including biogas production from biomass. Based on the techno-economic evaluation results, the biogas generation exhibits a longer dynamic payback period, 12.03 years, and a lower internal rate of return, 13.49%, when assessed using dynamic payback period and internal rate of return as evaluation metrics. When utilizing the net present value as the evaluation metric, the results revealed that biogas generation yields a substantial net present value of 11.94 million Chinese Yuan per megawatt. A study conducted by Meng et al. (2020) involved the assessment of various bioenergy initiatives that utilized crop straw on a large scale in China. The biogas project has the capacity to produce 4 × 10^6^ m^3^ of biogas annually, with a market value of 1.5 Chinese Yuan per m^3^. Consequently, the direct product benefit amounts to 0.405 million Chinese Yuan annually. Additionally, the project yields 300 tons of biogas digestate, which can be utilized as fertilizer, and has an estimated value of 100 Chinese Yuan per ton. Therefore, the annual profit of the by-product is 0.05 million Chinese Yuan.

In summary, a comprehensive economic and environmental assessment of a bioenergy project must include feedstock production and processing, energy conversion, and distribution. A proficient techno-economic evaluation must also include the local economic, social, and regulatory frameworks and involve stakeholders in decision making. The economic and technological components of bioenergy efforts must be assessed to determine their viability and sustainability. This method can improve energy infrastructure.

## Environmental impact assessment

Environmental impact assessment of sustainable rice straw utilization determines the potential consequences of using rice straw as a source of energy, e.g., biofuels. Through the environmental impact assessment, potential environmental impacts are identified and evaluated to ensure that any negative impacts are minimized or avoided. The assessment results will help determine the most environmentally responsible ways for using rice straw feedstock for energy generation. The assessment process for biogas as a biofuel and digestate as a fertilizer typically includes an examination of the following topics:

- **Greenhouse gas emissions**: While biofuels are often promoted to reduce greenhouse gas emissions, producing and using some biofuels can result in high emissions. For example, growing corn for ethanol production can require a large amount of fertilizer, resulting in nitrous oxide emissions, a potent greenhouse gas (Aristizábal-Marulanda et al. 2021). Moreover, the direct use of digestate as a fertilizer can reveal different types of emissions, especially nitrogenous emissions, such as ammonia, nitrous oxide, and nitric oxide (Pan et al. 2022).
- **Water resources depletion**: These crops require significant water for irrigation, which can pressure local water resources and reduce availability for other uses (Sreekumar et al. 2020). Various energy crops, such as sugarcane, maize, and oil palm, have comparatively high water needs at commercial production rates and, unless they can be irrigated, are best adapted to tropical regions with abundant rainfall (De Fraiture et al. 2008).
- **Land-use change**: Growing crops for biofuels can require substantial amounts of land, leading to the conversion of natural habitats, such as forests and wetlands. This can result in the loss of biodiversity and reduced carbon sequestration (Amezcua-Allieri et al. 2019). According to a study conducted by Elizabeth Marshall et al. (2011), sugarcane ethanol and soybean biodiesel, each contribute almost 50% of the anticipated indirect destruction of 121,970 km^2^ by 2020, resulting in a carbon debt that would take approximately 250 years to repay if these biofuels were used in place of fossil fuels. Furthermore, (Fargione et al. 2008) determined that if peatlands in Southeast Asia are turned into palm oil plantations for the production of biodiesel, which may take 423 years to repay the “carbon debt” incurred by the land-use change.
- **Air pollution**: Burning biofuels, e.g., biogas, can result in emissions of particulate matter and other air pollutants, which can have negative impacts on human health and the environment (Osman et al. 2021b). In this context, Huang et al. (2021) evaluated the effects of the emissions of global solid biofuel stoves on average particulate matter (PM2.5) and ozone air quality and their corresponding impacts on human health. The results revealed that over China, India, and sub-Saharan Africa, the annual average surface concentrations of particulate matter 2.5 are as high as 23.1 μg m^−3^ as a result of global solid-fuel stove emissions during the period 2006–2010. For the surface ozone, the global solid-fuel stove emissions led to up to 5.7 parts per billion by volume increases in surface ozone concentrations.

The most common method to conduct an environmental assessment is to use a life cycle assessment approach. Life cycle assessment works following the principles of the International Organization for Standardization (ISO 2006a, 2006b). The key processes of life cycle assessment span the whole life cycle of a certain product, starting from the extraction of raw material to manufacturing, distribution, usage, and disposal. Because a product cannot be created, made, or sold without components, materials, energy, and transportation, recognizing the primary environmental challenges across a product’s complete life cycle is complicated (Rebitzer et al. 2004). As a result, a systematic analytical technique can help understand these environmental challenges of products across their full life cycle, which is called the life cycle assessment method (Lee and Inaba 2004).

### Life cycle assessment description and implementation

The key processes span the whole life cycle of a product, from raw material extraction to manufacturing, distribution, usage, and disposal. Life cycle assessment is a systematic approach that permits the quantitative investigation of a product’s environmental burdens across the full life cycle from the cradle to the grave, which works following the principles of the International Organization for Standardization (ISO 2006a, 2006b).

The life cycle assessment was established in the USA in the 1970s, and since then, has become the most valuable and common tool in the world for quantifying and comparing the environmental impacts of products. Usually, life cycle assessment research is classified into two models, attributional and consequential life cycle assessment (Rehl et al. 2012). The attributional model gives a specific extent of the functional unit, indicating how the effect on the environment belongs to the product shares. In contrast, the consequence model forecasts the change in the functional unit, referring to how the environment is affected by the different processes of the product (Rebitzer et al. 2004). The life cycle assessment model consists of four main phases reported in the international organization for standardization series 14040/14044: (i) goal and scope definition, (ii) inventory analysis, (iii) impact assessment, and (iv) interpretation.

#### Goal and scope definition

**Fig. 8:**
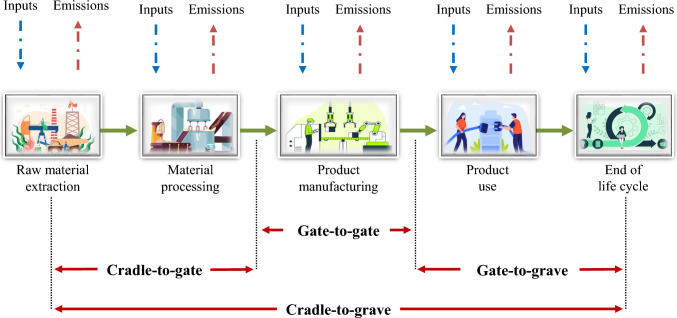
Models for life cycle assessment based on the scope of the analysis. Different models can be used in life cycle assessment studies according to the scope of evaluation and the availability of case data. Most studies use parietal models, such as gate-to-gate, cradle-to-gate, or gate-to-grave, for the same reasons. However, some comprehensive studies use the entire cradle-to-grave model to evaluate different processes or products, giving a clearer image of the associated environmental impacts

Determining the objective requires answering a number of fundamental issues, such as why we do the life cycle assessment. What are the objectives of conducting life cycle assessment studies? Whom are the intended recipients or target groups of the message or communication being conveyed? These questions relate to defining the main goal of the life cycle assessment studies (Lee and Inaba 2004). Defining the scope is connected to choosing the suitable functional unit, which is a distinctive aspect of life cycle assessment from other environmental assessment methods (Rebitzer et al. 2004). Moreover, life cycle assessment is accomplished by specifying the product scope of the system as models (ISO 2006a) that specify the system’s inputs as well as outputs (reference flows), as shown in Fig. 8.

#### Life cycle inventory analysis

Each operation within a product system necessitates the gathering of distinct data. The life cycle inventory pertains to the compilation of inputs and outputs that are associated with the function or product of a given process (ISO 2006b). The collection and aggregation of data is one of the most time-consuming tasks in life cycle assessment work and greatly affects the results if they are not accurate enough (Rebitzer et al. 2004). The absence of easily accessible inventory data continues to be a significant impediment to life cycle assessment implementation. However, various databases have been built over the last decades to ease life cycle inventory and reduce duplication of data gathering (Notarnicola et al. 2015). These databases include broad national or regional databases, industry databases, and consultant databases, frequently included as life cycle assessment software tools.

#### Life cycle impact assessment

The objective of the life cycle impact assessment is to convert the materials generated during the entire life cycle of a product into probable environmental burdens, with the purpose of identifying the various factors and consequences associated with the product (ISO 2006a). The life cycle impact assessment is used to quantify environmental impacts by multiplying the findings of the life cycle inventory (mass-environmental burdens/functional unit) by impact factors (de Bruijn et al. 2002a). The life cycle impact assessment is mostly composed of the following steps: (i) determination of the appropriate effect categories that are related to the studied product, (ii) classification of the fundamental flows according to their influence, and (iii) characterization with the use of conversion factors, where prospective impacts are modeled in order to generate an indication for the impact category (ISO 2006b). In this context, several life cycle impact assessment models are established to determine the mid-and end-point categories. One of the most common used is Centrum voor Milieuwetenschappen, known as CML 2001 model, which was developed by the Institute of Environmental Science, Leiden University, the Netherlands (de Bruijn et al. 2002a). CML 2001 model is used to determine the mid-point life cycle impact assessment categories (e.g., climate change, acidification, ozone layer depletion, and ecotoxicity) (Rebitzer et al. 2004).

#### Results interpretation

The conclusive stage of the life cycle assessment involves the interpretation of the outcomes obtained from the previous stages. This process entails the integration and evaluation of the results while considering the uncertainties inherent in the relevant data and the assumptions made during the study. The interpretation process should create results and give suggestions that abide by the objectives and limitations of the research by defining the goal and scope, as well as considering the suitability of the functional unit and the boundaries of the system (de Bruijn et al. 2002b). The results of life cycle assessment studies should be clearly presented to assist research users in evaluating their strengths and potential shortcomings in light of any recognized study limitations (Lee and Inaba 2004). The International Organization for Standardization series structured the interpretation step into three parts: (i) identifying important concerns based on life cycle inventory and life cycle impact assessment results, (ii) evaluating results for sensitivity, completeness, and consistency, and (ii) concluding, suggesting, and documenting these findings (ISO 2006a, 2006b).

**Fig. 9:**
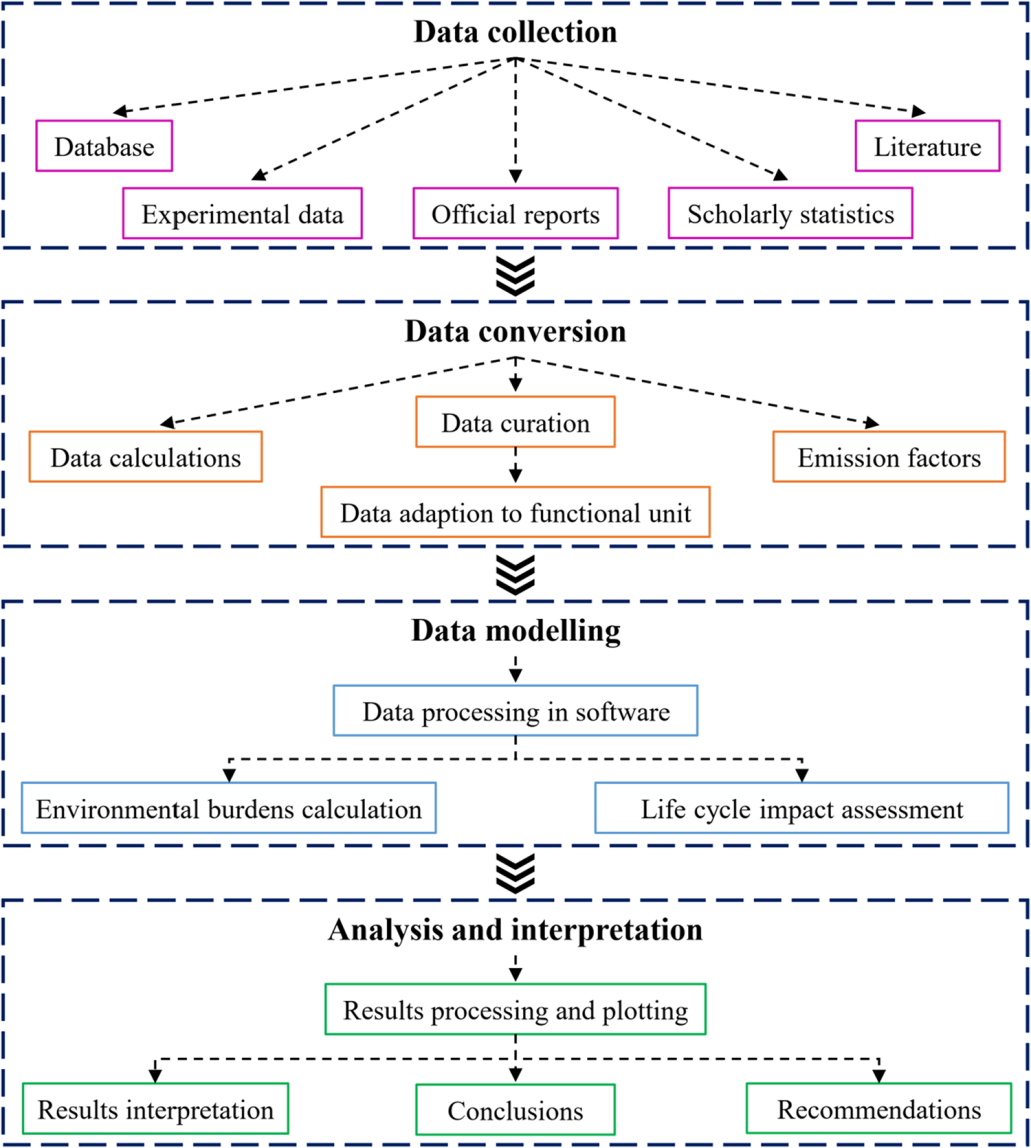
Schematic diagram of implementation steps of life cycle assessment model. The implementation of a life cycle assessment mainly involves four main steps; Collect data, transform data, process data, and interpret results. Data collection is the most critical step since collecting data needs a long time and intensive investigation. The other steps are less complicated than data collection, but each step has specific implementation requirements, such as the accuracy of data conversion and interpretation of results

The life cycle assessment model is typically constructed in multiple processes, including data collection, conversion, modeling, and data analysis and interpretation. Collecting data is a challenging stage of life cycle assessment because data accuracy determines the quality of obtained results. Data can be collected from various sources according to the case study, such as datasets, official reports, experimental data, and literature. After data collection, this data is managed by aggregation, curation, and adaptation. Then, data is processed in a life cycle assessment software to calculate the environmental burdens. Finally, the results are interpreted, and recommendations are given. The main implementation steps are illustrated in Fig. 9.

### Life cycle assessment research on rice straw utilization

Life cycle assessment research for rice straw valorization typically evaluates the environmental impact of converting rice straw into valuable materials or energy. This kind of research can include analyzing the inputs and outputs of various processes, such as composting, pyrolysis, or fermentation, as well as evaluating the overall energy efficiency and greenhouse gas emissions of these processes. As shown in Table 5, the most common approach for rice straw utilization is producing biofuels, e.g., biogas, biohydrogen, and bioethanol. Usually, these sustainable approaches are compared with conventional reference systems, such as straw open-field burning, fossil fuel-based energy production, or synthetic biofuels or gases. Most of the results revealed that using rice straw as a biomass can reduce the environmental impacts compared to other non-renewable types of biomass.

**Table 5:** Recent life cycle assessment studies on sustainable utilization of rice straw in China

Province	Life cycle assessment model	Life cycle impact assessment model	Functional unit	Studied scenarios	Main findings	References
Jiangsu	Cradle-to-gate	Not reported	1 megajoule syngas	Comparing synthetic natural gas production from rice straw with coal-based synthetic natural gas	Carbon dioxide emissions from rice straw-based synthetic natural gas were negative and lower than that of coal-based synthetic natural gas	Feng et al. 2017
Hubei	Field-to-gate	CML 2001	1-ton dry rice straw	Utilization of rice straw to produce three types of biofuels (biogas, syngas, briquette fuel)	The syngas scenario was the best sustainable option (net greenhouse gas -2,315 kg carbon dioxide equivalent/ton dry rice straw), followed by briquette fuel and biogas	Alengebawy et al. 2022c
Anhui, Jiangsu, and Shandong	Not reported	CML 2001	1 kg lignocellulosic crop wastes	A comparative life cycle assessment study to assess the treatment of three lignocellulosic crop wastes (rice straw, rice husk, and corn straw) for biofertilizer production using *Tenebrio molitor* Linnaeus larvae	The rice straw scenario had the lowest environmental effect, compared to other scenarios, for all impact categories except photochemical oxidation	He et al. 2021
Jiangsu, Jilin, Hebei, Shaanxi, and Sichuan	Cradle-to-gate	ReCiPe	1 ton dry crop straw	A comparative life cycle assessment was performed to determine the greenhouse gas emissions of the three crop straws (rice, maize, and wheat) treatment methods for biogas production, incineration, and livestock feed production	The maximum greenhouse gas emissions per unit area of household are 12,033.77 kg carbon dioxide equivalent/hectare in Jiangsu and 3599.77 kg carbon dioxide equivalent/hectare in Jilin (minimum)	Xu et al. 2022
Hubei	Gate-to-grave	CML 2001	1-ton digestate	Producing four types of biofertilizers from digestate (biofertilizer pellets, bio-compost, powder biofertilizer, and liquid biofertilizer)	The most sustainable treatment choice was liquid biofertilizer, followed by biofertilizer pellets, powder biofertilizer, bio-compost. All scenarios achieved environmental benefits compared to chemical fertilizer	Alengebawy et al. 2022b
Jilin	Cradle-to-grave	Not reported	1-ton straw	A comparison of three straw utilization scenarios; direct combustion for power generation, straw cement-bonded particleboard, and straw particleboard compared with straw open-field burning	The environmental impacts of the three scenarios are reduced compared to the reference system; straw combustion reduced greenhouse gas emissions by 30%	Shang et al. 2020
Hubei	Gate-to-gate	CML 2001	1 megajoule energy	Comparing three scenarios of biogas valorization; biogas upgrading, biogas boiler, and biogas combustion in a combined heat and power unit	The upgrading scenario achieved the highest environmental benefits, and the combined heat and power scenario was the second, followed by the boiler scenario	Alengebawy et al. 2022a
Jiangsu	Cradle-to-grave	Not reported	1000 kg biomethanol	A life cycle assessment to analyze the influence of biomethanol synthesis from rice straw on pollutant emissions	Global warming dominates the effect categories, and biomass-based methanol reduces global warming more than coal-based	Xiao et al. 2009

Rice straw as biomass shows promising results in terms of environmental impacts compared to conventional feedstocks, i.e., coal. Several studies assessed using rice straw for biorefinery approaches, mainly biogas, and the results revealed that rice straw could contribute significantly to emissions sinks. Regarding the life cycle assessment model, studies were conducted on different regions and cases in China, involving different scopes, functional units, and assessment methods, giving wide-range results to help construct Chinese inventory data

The names of life cycle impact assessment models, such as CML 2001 and ReCiPe, are given as they are provided by the developers

Moreover, most studies focused on evaluating greenhouse gas emissions, especially carbon dioxide, the major contributor to global warming and climate change problems. In terms of life cycle assessment models, the most commonly used functional unit is either the mass of straw as an input material, e.g., 1 kg or 1-ton biomass/straw, or the energy produced as an output, e.g., 1-megajoule energy. Furthermore, the partial model of life cycle assessment is commonly used, such as cradle-to-gate, gate-to-gate, and gate-to-grave, making the assessment process unable to comprehensively evaluate the entire process related to rice straw. Thus, addressing upstream and downstream processes via the cradle-to-grave model is essential to fully understand the emissions of each stage, which will help implement new strategies to reduce these emissions.

In summary, life cycle assessment measures the environmental implications of bioenergy initiatives, revealing their pros and cons. Life cycle assessment results might vary depending on assumptions and methods, as well as area economic, social, and regulatory conditions. To analyze the sustainability of bioenergy initiatives, life cycle evaluation must be used in conjunction with other sustainability measures, including social and economic sustainability.

## Perspective

The Chinese government has pledged to mitigate carbon emissions by 2030. In this context, promoting bioenergy, a technology with low, neutral, or even negative carbon emissions, is needed to attract full-scale plants (Liu et al. 2021b). This will not only contribute to carbon reduction but also to proper waste management of agricultural waste such as rice straw which is abundant in China and has been a matter of concern for efficient management. Traditionally, the anaerobic digestion of rice straw had been a problem due to the complex structure and solid-state mode of anaerobic digestion (Wang et al. 2020b). However, with the recent inventions of different pretreatment techniques, the problem has been significantly overcome. However, higher pretreatment cost still demotivates the stakeholders from adopting biogas production from rice straw. Efficient anaerobic digestion leading to better biogas production can offset the pretreatment cost. Therefore, research on making the anaerobic digestion of rice straw more efficient is imperative.

On the other hand, biogas produced should be used efficiently to improve the overall economy of the process. Traditionally, biogas was used for heating and cooking purposes by burning (Garfí et al. 2019). There is currently multifaceted use of biogas produced, and each use has merits and demerits. More research is required to suggest a better choice of use of biogas produced depending on several factors, such as cost of biogas cleaning, quality of biogas produced, maturity of technology, climatic and topographic conditions, and end use of biogas. This will help stakeholders choose the best option to maximize the efficiency of the overall process. Moreover, stakeholders should have ample information on equipment choice, which is mostly related to investment and maintenance costs, exhaust emissions, and reliability.

Digestate management is another issue that needs significant interest in the coming years. Traditionally, spreading on the field is common as digestate is rich in nitrogen and phosphorus (Vázquez-Rowe et al. 2015). However, this method is not opted for in many countries due to the issues of germ infection, especially if the rice straw is co-digested with manure. The cost of digestate transportation is also huge and needs to be reduced significantly. Drying of sludge is the common method currently adopted, but more research is in progress to extract valuable products from the sludge to support the circular economy (Peng et al. 2023). Therefore, more research on novel technologies is needed for valuable compound recovery, including but not limited to nutrients, minerals, and bioactive compounds. Moreover, developing novel techniques for the evaluation and delineation of digestate, with the objective of enhancing comprehension of its constitution and feasible applications.

## Conclusion

Bioenergy is renewable and eco-friendly, reducing greenhouse gas emissions. Biogas is crucial to a sustainable energy system and climate change mitigation. Therefore, the sustainable management of rice straw for bioenergy (biogas) and value-added by-products (biofertilizer) can achieve these goals. Biogas generation as a renewable energy source maximizes environmental and social advantages, coupling with proper biogas digestate management. Since the produced biofertilizers can help improve soil fertility and reduce the use of chemical fertilizers. Besides, using biogas for energy generation might also reduce climate change and lead to sustainability.

Additionally, mitigation measures such as source reduction, material recycling, and renewable energy production should be implemented in order to ensure that the sustainable management of rice straw is both beneficial to the environment and economically viable. Thus, the full bioenergy life cycle from feedstock production and processing to energy conversion and distribution must be included to assess the economic and environmental consequences. Life cycle assessment, techno-economic evaluation, and other sustainability criteria must be integrated to achieve a circular bioeconomy. Therefore, more green processes should be involved in the integrated approach of rice straw management.
